# What is the value and impact of quality and safety teams? A scoping review

**DOI:** 10.1186/1748-5908-6-97

**Published:** 2011-08-23

**Authors:** Deborah E White, Sharon E Straus, H Tom Stelfox, Jayna M Holroyd-Leduc, Chaim M Bell, Karen Jackson, Jill M Norris, W Ward Flemons, Michael E Moffatt, Alan J Forster

**Affiliations:** 1 Faculty of Nursing, University of Calgary, 2500 University Drive NW, Calgary, Alberta T2N 1N4, Canada; 2 Keenan Research Centre in the Li Ka Shing Knowledge Institute of St. Michael's Hospital, Toronto, Ontario, Canada; 3 Faculty of Medicine, University of Calgary, Calgary, Alberta, Canada; 4 Health Systems and Workforce Research Unit, Alberta Health Services, Calgary, Alberta, Canada; 5 Research and Applied Learning Division, Winnipeg Regional Health Authority, Winnipeg, Manitoba, Canada; 6 Department of Medicine, University of Ottawa, Ottawa Hospital Research Institute, Ottawa, Ontario, Canada

## Abstract

**Background:**

The purpose of this study was to conduct a scoping review of the literature about the establishment and impact of quality and safety team initiatives in acute care.

**Methods:**

Studies were identified through electronic searches of Medline, Embase, CINAHL, PsycINFO, ABI Inform, Cochrane databases. Grey literature and bibliographies were also searched. Qualitative or quantitative studies that occurred in acute care, describing how quality and safety teams were established or implemented, the impact of teams, or the barriers and/or facilitators of teams were included. Two reviewers independently extracted data on study design, sample, interventions, and outcomes. Quality assessment of full text articles was done independently by two reviewers. Studies were categorized according to dimensions of quality.

**Results:**

Of 6,674 articles identified, 99 were included in the study. The heterogeneity of studies and results reported precluded quantitative data analyses. Findings revealed limited information about attributes of successful and unsuccessful team initiatives, barriers and facilitators to team initiatives, unique or combined contribution of selected interventions, or how to effectively establish these teams.

**Conclusions:**

Not unlike systematic reviews of quality improvement collaboratives, this broad review revealed that while teams reported a number of positive results, there are many methodological issues. This study is unique in utilizing traditional quality assessment and more novel methods of quality assessment and reporting of results (SQUIRE) to appraise studies. Rigorous design, evaluation, and reporting of quality and safety team initiatives are required.

## Background

Over the last four decades, there has been a growing interest in improving the quality of care provided to patients. Recipients of care, providers, and healthcare leaders acknowledge that patient harm resulting from the delivery of healthcare is far more common and serious than they would like. For example, studies indicate that between 5% and 20% of patients admitted to hospital experience adverse events (AEs). AEs cost healthcare systems billions of dollars in additional hospital stays; retrospective reviews judge that between 36% and 50% of these AEs could have been avoided under different circumstances [1-4]. Building a culture of safety is cited as one of the most important aspects of improving patient safety and quality of care [5]. This requires an environment in which staff can speak freely about the lack of quality in the delivery of care, report errors, close calls, and hazardous situations that occur in the system, and feel empowered to implement changes that impact patient, provider, and system outcomes [6-8].

Quality and safety teams have been proposed as one strategy for professionals to discuss threats to quality and patient safety, and to identify and implement actions towards building safer systems [7,9]. These teams (often called quality improvement teams, quality collaboratives, clinical networks, or safety teams) are groups of individuals brought together to undertake specific initiatives to improve the quality of care [10]; care that is timely, effective, patient centred, efficient, equitable, and safe [11]. These team initiatives are often focused on designing and redesigning structures and/or processes of care at the local and system level, to yield better results for not only patients, but also providers and the broader health system [12]. If health organizations are to improve the quality of care and enhance patient safety, it is essential that there is a more in-depth understanding of how these teams are established, the barriers and facilitators to establishing and implementing teams and team initiatives, as well as the strength of the evidence about the impact of team initiatives.

Before embarking on a national study to survey and interview senior leaders and team members of quality and safety teams across Canada, a scoping review of the literature was undertaken to understand the types of quality and safe team initiatives, the evidence about their impact, and the barriers and facilitators to establishing teams and team initiatives.

## Methods

### Data sources and searches

We searched MEDLINE (1980-November 2007), EMBASE (1980-November 2007), CINAHL (1982-November 2007), Cochrane Effective Practice of Care, PsycINFO and ABI Inform (1980 to November 2007). Grey literature and websites were also searched. If a publication area could be identified on websites, this area was specifically searched rather than the entire site.

Combinations of the following search terms were used: patient safety, quality improvement, safety, quality, collaborative, team, committee, model, initiative, and clinical microsystems. Appropriate wildcards were used. Additional articles were identified through review of reference lists (see Additional file 1, Tables S1 and S2).

### Study selection

All abstracts were reviewed independently by multidisciplinary teams of two reviewers using the following inclusion criteria: qualitative or quantitative study; study occurred in an acute care centre; English language publication; description of how quality and safety teams were established, implemented and/or the impact of teams and their initiatives on provider, patient, and/or system outcomes; or description about barriers and/or facilitators to the establishment and implementation of quality and safety teams. Disagreements about inclusion were reviewed by two independent reviewers. Full text articles were retrieved and were further reviewed by two independent investigators. Disagreements between a set of reviewers were both reviewed and resolved by SES and DEW through consensus. Inter-rater agreement between reviewers was assessed using Cohen's k coefficient.

### Data abstraction and quality assessment

Initial data abstraction was performed by two independent reviewers, using a standardized data abstraction form (see Additional file 1, Table S3). Differences in abstraction between reviewers were resolved by a third reviewer.

The scoping review was designed according to recognized methodology [13], including a thorough documentation of the process for selection and inclusion of studies, data abstraction methods, traditional methodological critique [14], as well as other threats to internal and external validity. For randomized controlled trials (RCTs), criteria included method of randomization, allocation of concealment, blinding, protection from bias, assessment of outcomes, and description of sites. For observational studies, assessment included description of cohorts and assessment of outcomes among other items. Qualitative studies were assessed for evidence of appropriate sampling, adequate description, data quality, and theoretical and conceptual adequacy [15].

The Cochrane Effective Practice and Organisation of Care (EPOC) taxonomy for quality interventions [16] was adopted to aid in documenting quality improvement efforts undertaken by teams, and to explore which techniques lead to improved outcomes. Additionally, The Standards for Quality Improvement Reporting Excellence (SQUIRE) guidelines, described elsewhere [17], were also used to enhance the critique and capture rigor within the variations in reporting across published studies. Frequencies of the items and corresponding sections within SQUIRE checklist (see Additional file 1, Table S3) were used to determine coverage (*i.e*., yes or no) and thoroughness in the reporting of those items (*i.e*., good, fair, poor).

## Results

### Data synthesis

**Figure 1:**
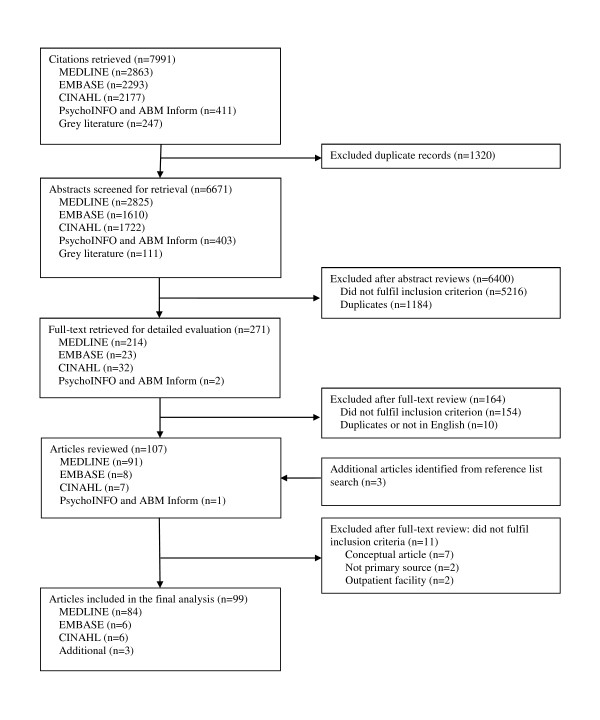
**Study selection process**.

After duplicates were removed from 7,994 citations retrieved, 6,674 abstracts were identified for review. Of these, 6,400 papers were excluded due to not meeting one or more of the inclusion criterion (Figure 1). Abstracts that did not describe teams in hospital settings, teams that did not undertake quality or safety work, or were not a quantitative or qualitative study were excluded. A total of 274 full-text papers were reviewed, and 99 papers were included within this review. Final inter-rater agreement reached 76.0% (Cohen's k coefficient *= *0.50). The heterogeneity of studies and outcomes/results reported precluded quantitative data analyses. Instead a descriptive summary is presented [13,18].

### Summary of research on quality and safety teams in acute care

To assist in the description and analysis, papers were categorized according to selected dimensions of quality defined by the IOM [11] (effectiveness, efficient, timely, patient centred, safety, equity; see Additional file 1, Table S4). Of the 99 papers included in our study, the primary focus of 45 addressed dimensions of effectiveness, 15 addressed aspects of efficiency, 16 focused on timeliness, 8 focused on patient centeredness, and 15 focused on safety. No papers focused on equitable care.

### Effectiveness papers

In 45 studies, the intent was to develop or utilize evidence about the impact of quality and safety teams and their initiatives. Quality initiatives were often focused on changes directed at clinical care processes for patient populations (*i.e*., maternity, cardiac, infection processes, asthma, and diabetes management) [19-44], exploration of effectiveness of quality and safety programs [45-49], and descriptions of team characteristics and leadership as important to the establishment, implementation, and/or outcome of initiatives [50-63].

Sixteen of the 45 quality initiatives [20-24,26-28,32-34,36,39,40,43,44] utilized best practice or national guidelines. Nine controlled studies reported statistically significant results [20,21,23,26,40,42,43,56,63], but only three studies reported statistically significant differences over a sustained period of time [20,23,56]. There were methodological flaws within the controlled studies, such as a greater dropout rate in the control group [56], and no description of case mix [20]. Horbar *et al*. [23] demonstrated the strongest design amongst the effectiveness papers. In a randomized trial, investigators tested whether teams in neonatal intensive care units exposed to a multifaceted collaborative QI intervention would decrease time to surfactant use after birth, and achieve improved patient outcomes for preterm infants of 23 to 29 weeks gestation. They reported a reduction in nosocomial infection (26% to 22%; p *= *0.007) and coagulase-negative staphylococcus infections (22% to 16.6%; p = 0.007) in neonates. Reduced rates were maintained over a four-year period.

### Patient-centred papers

Eight studies focused on improving and eliciting feedback about the patients' experience with programming and transitions in health systems (*i.e*., pain management programs, admission, and discharge processes). Bookbinder *et al*. [64], the only controlled study in this group, implemented a number of clinical care processes to improve palliative care for inpatients who were expected to die from advanced disease. Patients in intervention units were more likely to have a comfort plan in place (p < 0.0001) and do-not-resuscitate orders than the comparison units (p < 0.0001). Six studies were descriptive and did not have a control group [65-70]; each reported positive improvements over time (*i.e*., facilitated patient-centred care and assessment, patient satisfaction, excellent ratings of new discharge processes). Two studies reported statistically significant improvements from baseline [64,65], one of which maintained the desired outcomes over a period of six months or more [65].

### Safety papers

Of the safety papers (n *= *15) many focused on the reduction of AEs and/or errors (n *= *12). Initiatives focused on medication concerns [12,71-77], decreasing prescribing and administration error [12,71,73-75,78,79], reducing medical error, increasing overall error, and/or near miss reporting [12,71,72,75,77,80,81], among other issues [82,83]. Four studies employed statistical testing, and all reported statistically significant findings for desired outcomes when compared with baseline (*i.e*., increased reporting, decreased errors, and reduction of preventable adverse drug events) [12,72,73,75]. Common interventions included education sessions and audit/feedback. With the exception of Carey *et al*. [75], who utilized an interrupted time series design, the remaining study designs were descriptive or before and after case series.

### Timeliness papers

Sixteen papers were directed at improving structural and care processes such as decreased time to treatment, waiting times, length of stay [84-98], overcrowding, and patient flow [99]. While the majority of authors suggested positive improvement [85-100], only six studies used tests of significance [84,86-88,90,92]. Statistically significant improvements from baseline (*i.e*., decrease in delay of treatment [28,84,86,87,92], timely diagnosis [86-88,92]) were found for all six studies, but there were no reports of sustainability of outcomes. With the exception of Horbar *et al*. [84], the study designs were weak (before and after case series or historically controlled).

### Efficiency papers

Fifteen studies were directed at changing clinical practice patterns, outcomes, and system processes to address costs [100-107] and/or resource utilization (*i.e*., people and services) [102,105,106,108-114]. Three of the studies reported significant outcomes (*i.e*., decreased length of stay, reduced number of non-clinically indicated tests, decreased costs associated with personnel) when compared with baseline [102,103,112] or a control inpatient unit [102].

Few papers (n *= *6) [25,51-53,57,59] focused specifically on barriers and facilitators to establishing, implementing, and measuring the impact of quality and safety team initiatives. However, regardless of study aim, the role of leadership, organizational culture, and access to resources in supporting quality and safety were consistent messages in all the studies. A selection of team attributes, processes, and structures were also identified as important to implementation of initiatives (*e.g*., physician champions, expertise, understanding of roles on the team, time for meetings).

### General description of teams and their initiatives

Various professionals were represented on the teams, including nurses, physicians, and pharmacists. Approximately one-third of the teams also had representation from administrative and clinical leadership positions, as well as quality improvement experts. Statistical expertise was only reported in four studies. Twenty-one studies reported participation in a formal collaborative such as the IHI Breakthrough Series [12,20-22,44,45,57,65,72,85] and the Vermont Oxford Network [23,46,58,84].

A diverse number of quality improvement techniques/interventions were used in improvement initiatives. Teams used a mix of professional, financial, organizational, and regulatory quality interventions (see Table 1). Educational meetings (n *= *59), audit and feedback (n *= *30), and other quality improvement methodology (n *= *54) such as plan-do-study-act cycles (PDSA, n *= *15), and were frequently used. In addition to these professional interventions, teams often reported structural changes within organizations and provider oriented interventions.

**Table 1:** EPOC quality improvement strategies

	N	%
**Professional interventions**		
Educational meetings	59	59.6
Other quality improvement techniques (*i.e*., PDSA, process mapping flowcharts)	54	54.5
Audit and feedback	30	30.3
Distribution of educational materials	18	18.2
Educational outreach visits	12	12.1
Reminders	11	11.1
Marketing	10	10.1
Patient mediated interventions	5	5.1
Local consensus processes	4	4.0
Local opinion leaders	1	1.0
**Financial interventions**		
Provider oriented	9	
Provider salaried service	4	4.0
Provider incentives	3	3.0
Fee-for-service	1	1.0
Institution grant/allowance	1	1.0
Patient oriented	0	0.0
Other	3	3.0
**Organisational interventions**		
Provider oriented		
Clinical multidisciplinary teams	99	100.0
Case management	17	17.2
Continuity of care	16	16.2
Communication and case discussion between distant health professionals	12	12.1
Revision of professional roles	11	11.1
Satisfaction of providers with the conditions of work and its material and psychic rewards	11	11.1
Skill mix changes	10	10.1
Formal integration of services	6	6.1
Arrangements for follow-up	5	5.1
Patient oriented		
Presence and functioning of adequate mechanisms for dealing with client suggestions and complaints	12	12.1
Consumer participation in governance of healthcare organisation	1	1.0
Structural interventions		
Changes in physical structure, facilities and equipment	23	23.2
Changes in scope and nature of benefits and services	19	19.2
Changes in medical record systems	16	16.2
Presence and organisation of quality monitoring mechanisms	15	15.2
Staff organisation	9	9.1
Other	4	4.0
Changes in the setting/site of service delivery	2	2.0
Ownership, accreditation, and affiliation status of hospitals and other facilities	1	1.0
**Regulatory interventions**		
Management of patient complaints	4	4.0
Peer review	1	1.0

### Critical appraisal of methodological quality and reporting of studies

A controlled study design was used in twenty-three studies: interrupted time series (n *= *7) [20,24,37,38,75,82,85], controlled before and after (n *= *9) [19,21,23,26,27,56,64,112,113], RCT (n *= *2) [84,102], cohort (n *= *2) [39,40], and case-control studies (n *= *3) [41-43]. Twelve controlled studies utilized patient charts and administrative databases to measure outcomes. Limitations of the reporting of the studies included sparse information about the control sites, potential differences of baseline measurement, and lack of information about data collection processes and tools. Most studies used uncontrolled study designs (n *= *76): before-and-after case series (n *= *29) [12,22,28-32,57,63,65,71-74,79,80,87-91,98,99,103-106,109,115], historically controlled (n *= *6) [33-36,86,92], and descriptive (*i.e*., cross-sectional, correlational, survey, case-report; n *= *36) [44-49,52-55,58,60-62,66-68,70,76-78,81,83,93-97,100,101,107,108,110,111,114,116]. Five were qualitative-descriptive or mixed methods [25,50,51,59,69].

While subject to a number of single-group threats to internal validity, the overall methodological quality of studies was weak (see Table 2). Particularly, there were concerns of selection bias from few details about the patient populations, patient care units, and/or individual organizations involved in collaboratives. Other weaknesses included a lack of description about methods to ensure data quality and accuracy, reliance on team self-report measures, and a lack of documented questionnaire reliability and validity. While most reported 'significant' or 'very positive' improvements as a result of the intervention(s), only one-third employed appropriate statistical tests to determine if the interventions did make a difference.

**Table 2:** Methodological status of controlled studies

Study	Design	Methodological status	Commentary on potential bias
Horbar *et al*. [84] (2004)	Randomized controlled	Randomization (computer generated), allocation concealment (investigators, prior to intervention), baseline (13 of 14 measures similar, no statistical testing), blinding (statistician), ITT (done), follow-up (100%)	Voluntary participation in collaborative: 114/178 hospitals eligible participated.
Curley *et al*. [102] (1998)	Randomized controlled	Randomization (blocked), allocation concealment (NS), baseline (18 of 19 similar), blinding (NS), ITT (NS), follow-up (NS)	Used a convenience sample for one measure; controlled for potential covariates in analyses; questionable construct validity for provider satisfaction.
Carlhed *et al*. [26] (2006)	Controlled before	Allocation (matched then randomized), allocation concealment (controls), baseline (7 of 7 similar), blinding (controls), ITT (NS), follow-up (NS)	Intervention group hospitals self-selected, whereas control hospitals were hospitals that did not self-select; no group differences at baseline; registry had continuous monitoring; no reason to believe proposition of patients with contraindications systematically differed.
Doran *et al*. [56] (2002)	Controlled before	Allocation (participant preference, attempts to randomize), allocation concealment (NS), baseline (NS), blinding (external reviewers), ITT (NS), follow-up (time 1: 85%, time 2: 74%; higher control group attrition)	Selection: sample may be biased towards those who responded most quickly; measurement: unlikely, external reviewers blinded to group allocation and not part of study, reported methods to avoid bias; attrition/exclusion: differences between intervention group and those who withdrew, greater drop-out in the control group; gave description of sample, but did not compare group characteristics; performance: unlikely, analyses at team level.
Hermida and Robalino [19] (2002)	Controlled before	Allocation (matched then randomized), allocation concealment (NS), baseline (higher outcomes in intervention group), blinding (NS), ITT (NS), follow-up (NS)	
Howard *et al*. [21] (2007)	Controlled before	Allocation (matched, wait-list control), allocation concealment (NS), baseline (2 of 6 similar - controls, 5 of 6 similar - delayed comparison), blinding (NS), ITT (NS), follow-up (NS)	Provided information on non-responders; selection: self-selection, 43/58 participated, group differences at baseline; provide evidence against regression to the mean and selection bias in the wait-list controls; no information on quality of the data source.
Bookbinder *et al*. [64] (2005)	Controlled before	Allocation (location - unit type), allocation concealment (NS), baseline (3 of 21 similar), blinding (NS), ITT (NS), follow-up (NS)	Measurement: no baseline data; developed tools with interrater reliability; attrition bias: short survival of patients on the oncology unit; one tool could not completed: use was limited to 50 patients on intervention unit; selection: loss to follow up on comparison unit; performance: not possible to control for extraneous variables; referral to consultation team, exposure of staff to other educational offerings, cultural and leadership styles.
Brickman *et al*. [27] (1998)	Controlled before	Allocation (location - hospital, unclear if 'randomization' occurred), allocation concealment (NS), baseline (NS), blinding (NS), ITT (NS), follow-up (NS)	Performance: changing processes.
Horbar *et al*. [23] (2001)	Controlled before	Allocation (project participation), allocation concealment (NS), baseline (9 of 9 similar), blinding (NS), ITT (NS), follow-up (attrition in control)	Selection: self-selection of institutions.
Wang *et al*. [113] (2003)	Controlled before	Allocation (location - unit type), allocation concealment (NS), baseline (10 of 12 similar), blinding (NS), analyses (covariates), ITT (NS), follow-up (NS)	Selection: allocated by unit type, differences between groups on baseline characteristics and outcome measures, controlled for characteristics in analyses; clinical significance of differences in question; no attrition bias; performance: likely with different unit types being compared; source of inventory data quality is not known.
Isouard [112] (1999)	Controlled before	Allocation (location - hospital), allocation concealment (NS), baseline (3 of 3 similar), blinding (NS), analyses (no covariates), ITT (NS), follow-up (NS)	Selection: well defined criteria for selection for AMI.
Cable [37] (2001)	Interrupted time series	Data points (pre - 42-47 months/data points, post 22 to 27 months/data points), blinding (NS), analyses (ARIMA, switching replication), ITT (NS), follow-up (100%)	Measurement: change in catheterization tray, which affected catheterization events.
Berriel-Cass *et al*. [20] (2006)	Interrupted time series	Baseline (retrospective, NS case mix; pre - 7/8 months/data points, post - 23/24 months/data points), blinding (NS), analyses (pre-post comparisons), ITT (NS), follow-up (NS)	
Carey and Teeters [75] (1995)	Interrupted time series	Baseline (pre - 6 months/data points, post - 15 months/data points), blinding (NS), analyses (np charts, no inferential statistics), ITT (NS), follow-up (NS)	Selection/attrition: NA; performance/measurement: nurses may have increased reporting after training program, rather than the intervention being efficacious; unclear as to whether there was a change in intervention midway or after training program.
Harris *et al*. [38] (2000)	Interrupted time series	Baseline (pre - 3 years/6 data points, post - 3 years/6 data points), blinding (NS), analyses (no inferential statistics), ITT (NS), follow-up (NS)	Performance: physicians were already beginning to establish criteria before implementation; selection: no information about the sample.
Bartlett *et al*. [85] (2002)	Interrupted time series	Baseline (1. pre - 20 weeks/data points, post - 20 weeks/data points; 2. pre - 10 weeks/6 data points, post - 25 weeks/14 data points), blinding (NS), analyses (no inferential statistics), ITT (NS), follow-up (100%)	Selection/attrition: unlikely; measurement/performance: team-self and director-reported 'significant improvements', attempts to blind director to team identity.
Fox *et al*. [24] (2006)	Interrupted time series	Baseline (pre - 15 months/5 data points, post - 27 months/9 data points), blinding (NS), analyses (no inferential statistics), ITT (NS), follow-up (100%)	Time series controls for selection, but does not for history, instrumentation, and testing; no testing and instruments using review of charts; difficult to determine if there were any historical events that may have influenced results.
Allison and Toy [82] (1996)	Interrupted time series	Baseline (pre - 6 years/data points, post - 5 years/data points), blinding (NS), analyses (no inferential statistics), ITT (NS), follow-up (NS)	Measurement/instrumentation: unclear as to how some of the data was collected.
Halm *et al*. [40] (2004)	Cohort	Cohort (matched, separate pre- post cohorts, 30 of 37 similar), blinding (NS), ITT (NS), follow-up (NS)	Selection: acknowledges pre-post comparison of separate groupings of patients who met criteria of CAP; samples matched for age, race, sex, severity of diseases, co-morbidities, etc.
Berenholtz *et al*. [39] (2004)	Cohort	Cohort (different ICU types, baseline NS), blinding (NS), ITT (NS), follow-up (NS)	Selection: no description of population; may not have accounted for other confounding factors such as antibiotic use and location of catheter insertion.
Brown *et al*. [42] (2006)	Case-control	Cohort (prospective, case mix 3 of 4 similar, before-after comparisons), blinding (NS), analyses (regression)	Participants matched on post-data; performance: defined eras and care; selection bias: no loss to follow up, matched on most confounding variables; no masking regarding exposure and outcome.
Houston *et al*. [43] (2003)	Case-control	Cohort (matched - chart review, NS case mix), blinding (NS), analyses (no inferential statistics)	
Bromenshenkel *et al*. [41] (2000)	Case-control	Cohort (chart review, NS case mix; pre-post comparisons), blinding (NS), analyses (no inferential statistics)	No information on comparability of cases and controls for confounding variables, or if data collection was masked with regard to disease status of participant.
			

Abbreviations: NS *= *not specified, ITT *= *intention to treat, ARIMA *= *Autoregressive integrated moving average, ICU *= *intensive care unit.

Qualitative studies provided a description of purposive sampling of key informants and efforts to assure sampling adequacy. Only two authors [25,51] provided descriptions of the method of analysis. There was limited discussion of how researchers assured rigor; one author discussed member checking [33]. None of the qualitative studies addressed more than three methods to improve validity [117].

The EPOC classification of quality interventions [16] was utilized to examine whether specific types of improvement interventions lead to positive outcomes. All studies used two or more interventions in their initiatives; thus, it was difficult to make judgements regarding the unique or combined contribution of selected interventions on positive outcomes. Furthermore, within the studies there was a mix of improved outcomes and no change in the identified outcome. Papers seldom provided sufficient information to determine the mechanism of change, or details regarding the robustness of interventions. Beyond a narrative account of quality improvement efforts, additional inquiry regarding the weight of evidence for a particular technique was precluded by the heterogeneity in outcomes, design, and topics that quality and safety teams addressed in this scoping review.

Across the studies, authors seldom provided essential elements of SQUIRE reporting. More specifically, efforts to address a number of issues related to internal and external validity, or the validity and reliability of assessment instruments were documented in less than one-quarter of studies. Detailed information about training of data collectors and interviewers or data quality and accuracy were infrequently discussed. Few authors reported analyses that included effect size and power (n *= *14) or the distribution and management of missing data (n *= *10). Only one-half of the authors contextualized findings within existing literature. The weakest section of reporting across studies was planning of the interventions, with less than half of studies including any of the five elements outlined by SQUIRE. The study aim, abstract, background knowledge, and description of the local problem were uniformly addressed across all studies. Six exemplar studies reported at least three-quarters of all SQUIRE elements [33,39,40,56,65,69].

## Discussion

Over the past twenty years, there has been substantial growth in the number of quality improvement teams [7,8,59]. Under the direction of clinical or administrative leadership, teams have collectively directed their efforts to changing clinical and/or system processes and structures with the goal to improve patient, provider and system outcomes. This review revealed that the foci within each of the dimensions of quality, the interventions implemented by teams, the composition of teams, and the context in which initiatives occur were diverse. It was surprising to find that best evidence (*i.e*., best practice guidelines or national guidelines) or research-based evidence was not always utilized in these initiatives.

Few papers focused on barriers and facilitators to establishing and measuring the impact of quality and safety team initiatives, however, most researchers reported factors that they believed influenced the success of the teams. Many factors that were identified as facilitators (*i.e*., senior leadership support, supportive organizational cultures, resources, ability to work as a team, physician 'opinion' leaders) are attributes of effective teams [118]. Often, these factors were identified as barriers if they were absent. Teams' perception of their success or failure often revolved around these factors. These findings are consistent with other authors [119-121] who have emphasized that strategic direction and vision of senior leadership, organizational culture, and support of leadership to remove barriers for teams are key to making a difference in quality and safety in organizations.

We found a lack of evidence about the attributes of successful and unsuccessful team initiatives, descriptions of how to establish and implement the teams, the unique or combined contribution of selected interventions, and the cost-benefit analyses of such initiatives. Future research could focus on the behaviours and actions of participants themselves, such as what actions senior leaders did to assure the team was successful and what role physicians and nurse champions played in winning the support of their colleagues [18].

We noted few methodologically strong studies. As a result, it is difficult to know whether the 'success' or 'failure' of quality and safety team initiatives are the result of the attributes and ideal mix of team members, team processes, period over which the initiatives occurs, certain clinical conditions and system processes, selected or combined interventions, the outcomes measured, or context in which the interventions occur. Understanding the unique and combined contributions of quality improvement interventions will require the use of rigorous designs and synthesis of study results through a systematic review. A broad-based scoping review does not seek to synthesize or weight evidence from various studies [13].

Despite this lack of evidence about the mechanisms by which intervention components and contextual factors may influence the study outcomes, quality improvement methodologies and quality collaboratives are popular methods for understanding and organizing quality improvement and safety efforts in hospitals. The nature of quality improvement is pragmatic; an examination of the 'real world.' Health systems are living laboratories that are complex, frequently unpredictable, and change is often multifaceted. Unfortunately, RCTs are often not an option and control groups may not be possible to understand localized microsystem or mesosystem change. However, moving away from weaker study designs (*e.g*., before and after designs) to designing evaluation of change initiatives that utilize more robust designs (*e.g*., interrupted time series or step wedge design) would enhance the science of quality improvement as well as strengthen the evidence about the actual effectiveness of methods used in initiatives.

Healthcare providers, senior leaders, and boards strongly affirm the importance of improving processes for assuring quality and safety, and require access to the best evidence to help achieve that goal. We observed that many documented improvements, and identified 'successes' have been reported using percentage changes over time without comparisons to control groups or subject to statistical testing. There needs to be more rigorous evaluation of the interventions to propose legitimately that 'evidence-based' practices be accepted. Considerable resources are allocated to changes associated with these initiatives. The time has come to decide whether this investment is justified.

Mittman [122] proposes that researchers, users, and stakeholders engage in rigorous evaluation and creation of a valid, useful knowledge and evidence base for quality and safety. This will require improved conceptions of the nature of quality and safety issues, an understanding of the mechanisms by which various structures and processes (*e.g*., quality improvement interventions) impact outcomes, stronger designed studies (*i.e*., time series), reliable and valid measurements, data quality control, and statistical processes to evaluate the impact of initiatives [123].

A strength of this review was the quality appraisal of reporting excellence using the newly established SQUIRE guidelines. Ogrinc *et al*. [17] have called for excellence in reporting as a means to share organizational learning and benefit care delivery. Our review revealed that the quality of current reporting varies widely. Improving the rigor of study methods and the reporting of study findings will build a stronger foundation and more convincing argument for future studies and the practice of quality improvement and safety in healthcare.

Limitations should be considered in interpreting the results of this review. First, the search was broad and included studies of quality and safety team initiatives without operational definitions of quality and safety. This may have introduced misclassification of the studies. However, we believe our selection process of an independent review by two investigators and unresolved disagreements on inclusion referred to a team of two reviewers strengthened our classification. Second, this review only addressed studies conducted in an acute care setting, thus results may not be applicable to outpatient and community settings.

## Conclusions

Clearly, there is much needed improvement in the design and reporting of quality and safety initiatives. If readers are to judge the internal and external validity of a study, investigators must provide enough information for critical appraisal of the intervention procedures, measurements, subject selection, analysis, and the context of the individual, group, organization, and system characteristics in which the intervention occurs. Knowing how the contextual factors compare to one's own circumstances is key to determining the generalisability and relevance of the results [124].

## Competing interests

The authors declare that they have no competing interests.

## Authors' contributions

DEW is the guarantor for the paper. DEW led the review, obtained funding for the study, and identified the research question. DEW and SS designed the search strategy. DEW, SES, HTS, JMH, CMB, KJ, WWF, MEM, and AJF screened search results and reviewed papers against the inclusion criteria. DEW, SES, and JMN extracted data and assessed papers for methodological and reporting quality. DEW and JMN synthesized the results, analysed the findings, and drafted the manuscript. All authors made critical revisions of the manuscript for intellectual content and approved the final version.

## Supplementary Material

Additional file 1 **Tables S1 to S4**. Table S1- Search strategies by database; Table S2- Distribution of references by electronic bibliographic source; Table S3- Data abstraction form; Table S4- Reviewed studies, differentiated by quality dimension. Click here for file
