# Supplementary material for: What is the value and impact of quality and safety teams? A scoping review
Source: Implement Sci. 2011 Aug 23;6:97. doi: 10.1186/1748-5908-6-97 (PMC3189393; doi:10.1186/1748-5908-6-97)
Supplement: Additional file 1 — Tables S1 to S4. Table S1- Search strategies by database; Table S2- Distribution of references by electronic bibliographic source; Table S3- Data abstraction form; Table S4- Reviewed studies, differentiated by quality dimension. [file 1748-5908-6-97-S1.DOC]

# Additional file 1

Table S1 – Search strategies by database

### Table S2- Distribution of references by electronic bibliographic source

### Table S3- Data abstraction form

Table S4- Reviewed studies, differentiated by quality dimension

### Table S1 – Search strategies by database

| **MEDLINE** (1980 to November Week 2 2007; combined with Cochrane EPOC RCT filter) |
| --- |
| 1 ((patient adj safety) and (framework? or model? or initiative?)).tw.  2 (quality adj improvement adj (framework? or model? or initiative?)).tw.  3 ((safety or quality) adj2 (collaborative? or team? or committee?)).tw.  4 (Total Quality Management/ or Quality Assurance, Health Care/) and (collaborative? or team? or committee?).tw.  5 exp Safety/ and exp Patients/  6 (clinical adj microsystem?).tw.  7 or/1-6  8 randomized controlled trial.pt.  9 controlled clinical trial.pt.  10 intervention studies/  11 experiment$.tw.  12 (time adj series).tw.  13 (pre test or pretest or posttest or post test).tw.  14 random allocation/  15 impact.tw.  16 intervention?.tw.  17 chang$.tw.  18 evaluation studies/  19 evaluat$.tw.  20 effect?.tw.  21 comparative study.pt.  22 or/8-21  23 Animals/ not (Animals/ and Human/)  24 22 not 23  25 7 and 24  26 limit 25 to yr="1980 - 2007" |
| **EMBASE** (1980 to 2007 Week 48; combined with Cochrane EPOC RCT filter) |
| 1 ((patient adj safety) and (framework? or model? or initiative?)).tw.  2 (quality adj improvement adj (framework? or model? or initiative?)).tw.  3 ((safety or quality) adj2 (collaborative? or team? or committee?)).tw.  4 Total Quality Management/ and (collaborative? or team? or committee?).tw.  5 exp Safety/ and exp Patient/  6 (clinical adj microsystem?).tw.  7 or/1-6  8 Randomized controlled trial/  9 random$.tw.  10 experiment$.tw.  11 (time adj series).tw.  12 (pre test or pretest or post test or posttest).tw.  13 impact.tw.  14 intervention?.tw.  15 chang$.tw.  16 evaluat$.tw.  17 effect?.tw.  18 compar$.tw.  19 (controlled adj study).tw.  20 or/8-19  21 Nonhuman/  22 20 not 21  23 7 and 22  24 limit 23 to yr="1980 - 2007" |
| **CINAHL (**1982 to November Week 5 2007; combined with Cochrane EPOC RCT filter) |
| 1 ((patient adj safety) and (framework? or model? or initiative?)).tw.  2 (quality adj improvement adj (framework? or model? or initiative?)).tw.  3 ((safety or quality) adj2 (collaborative? or team? or committee?)).tw.  4 exp Quality Assurance/ and (collaborative? or team? or committee?).tw.  5 exp Safety/ and exp Patients/  6 (clinical adj microsystem?).tw.  7 or/1-6  8 clinical trials/  9 (controlled adj (study or trial)).tw.  10 (randomised or randomized).tw.  11 (random$ adj1 (allocat$ or assign$)).tw.  12 comparative studies/  13 experiment$.tw.  14 (time adj series).tw.  15 impact.tw.  16 intervention?.tw.  17 evaluat$.tw.  18 effect?.tw.  19 exp pretest-posttest design/  20 exp quasi-experimental studies/  21 or/8-20  22 7 and 21 |
| **PsycINFO** and **ABI Inform** (1980 to November 2007) |
| (KW=(("patient safety") or ("quality improvement")) and KW=(framework* or model* or initiative*))  OR  (KW=("clinical microsystem*" or "safety collaborative" or "safety team" or "safety committee" or "safety collaboratives" or "safety teams" or "safety committees" or "quality collaborative" or "quality team" or "quality committee" or "quality collaboratives" or "quality teams" or "quality committees")) |
|  |

**Table S2-** Distribution of references by electronic bibliographic source

| **Database** | **Host** | **Dates covered** | **Date searched** | **Hits** |
| --- | --- | --- | --- | --- |
| MEDLINE | Ovid | 1980 – November 2007 | 6 December 2007 | 2863 |
| EMBASE | Ovid | 1980 – November 2007 | 6 December 2007 | 2293 |
| CINAHL | EBSCO | 1982 – November 2007 | 6 December 2007 | 2177 |
| PsycINFO  ABI/Inform | Scholars Portal | 1980 – November 2007 | 6 December 2007 | 411 |
| Google | Internet | 17-18 December 2007 | 17-18 December 2007 | 70 |
| Intute | Internet | 17-18 December 2007 | 17-18 December 2007 | 0 |
| Agency for Healthcare Research and Quality | Internet | 18 December 2007 | 18 December 2007 | 173 |
| Medical Research Council (UK) | Internet | 18 December 2007 | 18 December 2007 | 0 |
| National Health and Medical Research Council (Australia) | Internet | 18 December 2007 | 18 December 2007 | 3 |
| National Health Service (UK) | Internet | 18 December 2007 | 18 December 2007 | 0 |
| Institute for Healthcare Improvement | Internet | 18 December 2007 | 18 December 2007 | 1 |
| U.S Department of Veteran Affairs | Internet | 18 December 2007 | 18 December 2007 | 0 |
| Reference list scan |  |  |  | 3 |
|  |  |  |  |  |

**Table S3-** Data extraction form

| ***Reference*** | |
| --- | --- |
| First author | |
| Title | |
| Journal reference | |
| ***Inclusion Criteria*** | |
| English language article | |
| Qualitative or quantitative study | |
| Type | |
| At least one of the following:  a) Description of establishment of quality and safety team/ or initiative /program/collaborative  b) Description of barriers and facilitators to establishment of initiative /program/collaborative  i) Barriers (describe)  ii) Facilitators (describe)  c) Description of implementation of quality and safety team initiative /program/collaborative | |
| Study in an acute care hospital | |
| ***Description of Study*** | |
| Design (use Cochrane Study Design checklist) | |
| Purpose of Study | |
| Objective measurement of impact | |
| Relevant and interpretable data presented or obtainable | |
| Study targets description of impact of initiatives or interventions on any outcome | |
| ***Study Population*** | |
| Inclusion criteria | |
| Exclusion criteria | |
| Demographics:  a) Mean age (range)  b) Female  c) Ethnicity  d) Co-morbid conditions | |
| Geographical Location | |
| Clinical Setting | |
| Intervention description | |
| Control description | |
| ***Qualitative Study (any study design)*** | |
| Evidence of appropriate sampling | |
| Adequate description of teams, experiences of being part of team, facilitators and barriers, perception of impact of teams, description of initiative | |
| Data quality | |
| Description of qualitative design | |
| ***Team Composition*** | |
| Consumer involved | |
| **Participants on the team** | |
| Professions:  a) Physician  b) Nurses  c) Other providers | |
| Level of training:  a) in training  b)licensed/accredited  c) mixed  d) clear | |
| Areas of expertise:  a) Medicine  b) Pediatrics  c) Quality improvement  d) Pharmacy  e) Administration  f) Other  g) Clear | |
| Number of participants | |
| **Team characteristics:**  a) Age (mean)  b) Race/ethnicity  c) Gender  d) Clear | |
| **Setting of initiative:**  a) Outpatient  b) Inpatient  c) Mixed | |
| **Academic status:**  a) University based teaching  b) Non teaching  c) Clear | |
| ***Description of Initiative/Program/Collaborative Included*** | |
| Assessment of Patients | |
| Medication | |
| Intravenous care | |
| Flow of Information | |
| Adoption and utilization of best practice guidelines/ care paths | |
| Discharge Planning | |
| Other | |
| ***Implementation of QI Initiative/Program/Collaborative or identified intervention*** | |
| Education/Teaching Sessions/Seminars /Inservices | |
| Audit and feedback | |
| Process of Care/Tools Best Evidence Guidelines Care Path | |
| PDSA cycles | |
| Other | |
| ***Implementation of Quality and Safety Team Initiative/Program/Collaborative Involved*** | |
| Patient care unit (s):  a) How many units  b) Type of unit | |
| Other hospital departments:  a) Pharmacy  b) Blood bank  c) Radiology  d) Other | |
| Hospital (s):  a) How many  b) Number of cities/regions within states or provinces  c) Number of states | |
| **Patient population:**  a) Respiratory  b) Cardiac  c) Gastroenterology  d) Infectious diseases  e) Other  f) Clear  g) Inclusion  h) Exclusion | |
| Patient characteristics:  a) How many patients involved in the initiative  b) Age  c) Gender  d) Ethnicity  e) Other | |
| **Providers of care:**  a) How many  b) Description | |
| **Country** | |
| **Type of collaborative (use Collaboratives checklist)** | |
| Length of intervention/ initiative/collaborative | |
| ***Outcomes (Results)*** | |
| Primary Outcome | |
| Secondary Outcomes | |
| **Limitations** | |
| ***SQUIRE Checklist*** | |
| **Title and Abstract** |  |
| 1 Title | (a) Indicates the article concerns the improvement of quality (broadly defined to include the safety, effectiveness, patient centeredness, timeliness, efficiency and equity of care) |
|  | (b) States the specific aim of the intervention |
|  | (c) Specifies the study method used (for example, ‘‘A qualitative study,’’ or ‘‘A randomised cluster trial’’) |
| 2 Abstract | Summarises precisely all key information from various sections of the text using the abstract format of the intended publication |
| **Introduction** |  |
| 3 Background knowledge | Provides a brief, non-selective summary of current knowledge of the care problem being addressed, and characteristics of organisations in which it occurs |
| 4 Local problem | Describes the nature and severity of the specific local problem or system dysfunction that was addressed |
| 5 Intended improvement | (a) Describes the specific aim (changes/improvements in care processes and patient outcomes) of the proposed intervention |
|  | (b) Specifies who (champions, supporters) and what (events, observations) triggered the decision to make changes, and why now (timing) |
| 6 Study question | States precisely the primary improvement-related question and any secondary questions that the study of the intervention was designed to answer |
| **Methods** |  |
| 7 Ethical issues | Describes ethical aspects of implementing and studying the improvement, such as privacy concerns, protection of participants’ physical wellbeing and potential author conflicts of interest, and how ethical concerns were addressed |
| 8 Setting | Specifies how elements of the local care environment considered most likely to influence change/improvement in the involved site or sites were identified and characterised |
| 9 Planning the intervention | (a) Describes the intervention and its component parts in sufficient detail that others could reproduce it |
|  | (b) Indicates main factors that contributed to choice of the specific intervention (for example, analysis of causes of dysfunction; matching relevant improvement experience of others with the local situation) |
|  | (c) Outlines initial plans for how the intervention was to be implemented—for example, what was to be done (initial steps; functions to be accomplished by those steps; how tests of change would be used to modify intervention) and by whom (intended roles, qualifications, and training of staff) |
| 10 Planning the **study** of the intervention | (a) Outlines plans for assessing how well the intervention was implemented (dose or intensity of exposure) |
|  | (b) Describes mechanisms by which intervention components were expected to cause changes, and plans for testing whether those mechanisms were effective |
|  | (c) Identifies the study design (for example, observational, quasi-experimental, experimental) chosen for measuring impact of the intervention on primary and secondary outcomes, if applicable |
|  | (d) Explains plans for implementing essential aspects of the chosen study design, as described in publication guidelines for specific designs, if applicable (see, for example, www.equator-network.org) |
|  | (e) Describes aspects of the study design that specifically concerned internal validity (integrity of the data) and external validity (generalisability) |
| 11 Methods of evaluation | (a) Describes instruments and procedures (qualitative, quantitative or mixed) used to assess  (i) the effectiveness of implementation,  (ii) the contributions of intervention components and context factors to effectiveness of the intervention  (iii) primary and secondary outcomes |
|  | (b) Reports efforts to validate and test reliability of assessment instruments |
|  | (c) Explains methods used to assure data quality and adequacy (for example, blinding; repeating measurements and data extraction; training in data collection; collection of sufficient baseline measurements) |
| 12 Analysis | (a) Provides details of qualitative and quantitative (statistical) methods used to draw inferences from the data |
|  | (b) Aligns unit of analysis with level at which the intervention was implemented, if applicable |
|  | (c) Specifies degree of variability expected in implementation, change expected in primary outcome (effect size) and ability of study design (including size) to detect such effects |
|  | (d) Describes analytical methods used to demonstrate effects of time as a variable (for example, statistical process control) |
| **Results** |  |
| 13 Outcomes | (a) Nature of setting and improvement intervention  (i) Characterises relevant elements of setting or settings (for example, geography, physical resources, organisational culture, history of change efforts) and structures and patterns of care (for example, staffing, leadership) that provided context for the intervention  (iii) Documents degree of success in implementing intervention components  (iv) Describes how and why the initial plan evolved, and the most important lessons learned from that evolution, particularly the effects of internal feedback from tests of change (reflexiveness) |
|  | (b) Changes in processes of care and patient outcomes associated with the intervention  (i) Presents data on changes observed in the care delivery process  (ii) Presents data on changes observed in measures of patient outcome (for example, morbidity, mortality, function, patient/staff satisfaction, service utilisation, cost, care disparities)  (iii) Considers benefits, harms, unexpected results, problems, failures  (iv) Presents evidence regarding the strength of association between observed changes/improvements and intervention components/ context factors  (v) Includes summary of missing data for intervention and outcomes |
| **Discussion** |  |
| 14 Summary | (a) Summarises the most important successes and difficulties in implementing intervention components, and main changes observed in care delivery and clinical outcomes |
|  | (b) Highlights the study’s particular strengths |
| 15 Relation to other evidence | Compares and contrasts study results with relevant findings of others, drawing on broad review of the literature; use of a summary table may be helpful in building on existing evidence |
| 16 Limitations | (a) Considers possible sources of confounding, bias or imprecision in design, measurement, and analysis that might have affected study outcomes (internal validity) |
|  | (b) Explores factors that could affect generalisability (external validity)—for example, representativeness of participants; effectiveness of implementation; dose-response effects; features of local care setting |
|  | (c) Addresses likelihood that observed gains may weaken over time, and describes plans, if any, for monitoring and maintaining improvement; explicitly states if such planning was not done |
|  | (d) Reviews efforts made to minimise and adjust for study limitations |
|  | (e) Assesses the effect of study limitations on interpretation and application of results |
| 17 Interpretation | (a) Explores possible reasons for differences between observed and expected outcomes |
|  | (b) Draws inferences consistent with the strength of the data about causal mechanisms and size of observed changes, paying particular attention to components of the intervention and context factors that helped determine the intervention’s effectiveness (or lack thereof) and types of settings in which this intervention is most likely to be effective |
|  | (c) Suggests steps that might be modified to improve future performance |
|  | (d) Reviews issues of opportunity cost and actual financial cost of the intervention |
| 18 Conclusions | (a) Considers overall practical usefulness of the intervention |
|  | (b) Suggests implications of this report for further studies of improvement interventions |
| **Other** |  |
| 19 Funding | Describes funding sources, if any, and role of funding organisation in design, implementation, interpretation and publication of study |
|  |  |

**Table S4- Reviewed studies, differentiated by quality dimension**

| **Study (Year)**  **Country** | **Dimension of Quality** | **Topic**  **Purpose** | **Design** | **Acute care centre (n); control (n)**  **Unit (n); control (n)**  **Sample** | **Primary outcomes** |
| --- | --- | --- | --- | --- | --- |
| Cheah ^33^ (2000)  Singapore | **Effective** | Cardiac (AMI patient care)  To evaluate the impact of a clinical pathway on the quality of care for patients admitted for uncomplicated AMI | Historically controlled | Hospital (1)  ICU, emergency (2)  pre n=100, post n=169 patients | **System:** Average LOS reduced (16.5%, p<.001).  **Clinical:** NS: readmission or complication rates. |
| Carlhed et al. ^25^ (2006)  Sweden | **Effective** | Cardiac (AMI treatment guidelines)  To evaluate to which extent a multicenter QI collaborative, in combination with local and regularly generated real-time feedback reports, can increase adherence to national AMI guidelines | Controlled before and after | Hospitals (19;19)  ICU - coronary care (19); ICU - coronary care (19)  intervention: n=3786 patients  control: n=2940 patients | **Clinical:** Intervention group improved across all 5 of indicators (p<.001), control group improved in treatment levels of clopidogrel (p<.001). Intervention group had greater improvements over time (mean absolute rates) for angiotensin converting enzyme inhibitors (1.4% vs. 12.6%, p=.002), lipid-lowering therapy (2.3% vs. 7.2%, p=.065), clopidogrel (26.3% vs. 41.2%, p=.010), heparin/low–molecular weight heparin (5.3% vs. 16.3%, p=.010), coronary angiography (6.2% vs. 16.8%, p=.027), lipid-lowering therapy (p=NS). |
| Cable ^36^ (2001)  USA | **Effective** | Cardiac (procedures)  To reduce the percentage of bilateral cardiac catheterizations in three catheterization laboratories | Interrupted time series | Hospital (3)  Catheterization laboratory (3) | **General:** by using interrupted time series design, accuracy and causal interpretability of the findings were considerably improved from original design. |
| Brush et al. ^27^ (2006)  USA | **Effective** | Cardiac (procedures)  To examine outcomes measured before and after implementation of a CQI program for cardiac procedures at a large complex community hospital | Before and after case series | Non-teaching community hospital (1)  Surgery - cardiac (1)  pre n=1511, post n=1943, follow-up n=2041 patients | **System:** Sustained physician and administration participation and buy-in.  **Clinical:** percutaneous coronary intervention: number of patients who received stents increased (23%, 83%, 81%, p<.05), IIb/IIIa antagonists increased (0%, 92%, 96%, p<.01); in-hospital mortality trend toward improvement (1.5%, 1.1%, 0.8%, p=NS); coronary artery bypass graft increased (3%, 1%, 1.4%), angiographic success (94%, 97% 95%; p<.001). |
| Blaylock ^34^ (1996)  USA | **Effective** | Cervical collars  To decrease the incidence of pressure ulcers related to the use of cervical collars | Historically controlled | Hospital (1)  Trauma (1)  pre n=61, post n=20 patients | **Clinical:** educational intervention: rate of pressure ulcers did not change; new brand of cervical collars intervention: patients did not exhibit tissue irritation or breakdown |
| Pierre ^30^ (2005)  USA | **Effective** | Clinical practice/ process (delirium prevention)  To improve detection, management, and prevention of delirium among older patients in hospitals | Before and after case series | Teaching hospital (1)  Medical (1)  pre n=2298, post n=1794 patients | **Clinical:** targeted medication use reduced (57%); small increase in the use of some drugs at follow-up; chlordiazepoxide and diazepam use increased at follow-up. Pharmacists rated 39%physician compliance for targeted-drug prescribing practices. |
| Bromenshenkel et al. ^40^ (2000)  USA | **Effective** | Clinical practice/ process (postoperative ileus)    To reduce the number of patients at risk for developing a postoperative ileus | Case-control | Hospital (1)  NR  case n=22 (ileus), control n=30 patients (no ileus) | **Clinical:** overall ileus rate decreased (4.8% to1.9%). |
| Cerulli & Malon ^28^ (2000)  USA | **Effective** | Clinical practice/ process (total parenteral nutrition)  To determine the impact of and compliance to a revised total parenteral nutrition order form | Before and after case series | Teaching hospital (1)  NR  n=50 orders | **Clinical:** total calories received on day 1 reduced (p<.007); dextrose concentrations reduced (5.1 to 3.7 g/kg per day, p =.004); hyperglycemia incidence (p=NS). |
| Newtown et al. ^24^ (2007)  Australia | **Effective** | Collaboratives  To identify and explore the barriers and facilitators to the implementation of the collaborative method at a single site participating in a statewide collaborative- heart failure disease management. | Qualitative, descriptive, mixed methods | Health care system (1)  NR | **Barriers/ facilitators:** organizational factors, team composition, dynamics and networking, changing doctor behavior, clinical leadership and communication. |
| Mills & Weeks ^56^ (2004)  USA | **Effective** | Collaboratives  To identify the organizational, interpersonal, and systemic characteristics of successful improvement teams | Before and after case-series | Medical QI teams (134)  NR | **Team:** 57% of teams were rated successful (> 20% improvement from baseline, for at least two months before the collaboratives end). Baseline: high performing medical quality improvement teams perceive their work to be part of their organization’s key strategic goals (p = .0425), their team members know and respect each other (p = .018), have worked together before (p = .022). Post: high performing teams have stronger team leadership (p = .029). |
| Ayers et al. ^58^ (2005)  UK, USA, Sweden | **Effective** | Collaboratives  To identify and synthesize characteristics of successful data-driven QI learning collaboratives | Qualitative, descriptive | Collaboratives (10)  NR | **Team:** successful collaboratives demonstrated: cultivating trust, attendance to the human dimension, nonlinear development, attendance to organizational culture, integrated philosophy of quality improvement, and a focus on process and outcome measurement to drive change. |
| Howard et al. ^20^ (2007)  USA | **Effective** | Coordination (organ donation)  To encourage adoption of best practices for identifying potential donors and obtaining consent for deceased organ donation | Controlled before and after | University-based and non-teaching hospitals (95; 125/19)  NR | **System:** increased proportion of eligible donors who became actual donors (60% vs. 51%, p<.001). |
| Irvine et al. ^62^ (2000)  Canada | **Effective** | Culture/ teams  To examine the relationship between participation on QI teams and changes in behavior consistent with QI principles; explore the relationship between team and attitudinal variables | Before and after case series | Hospitals (4)  NR  n=79 team members | **Team:** increase in empowerment over time (7.84 to 8.08, p<.05); post-team organizational commitment is influenced by team success, the relationship which is conditional upon the individual's identification with the team; organizational citizenship behavior is influenced by perceptions of team success, but only when the individual identifies with team; support for conditional relationship between perceived team success, team identification, and behavioral outcome; empowerment had direct effect on job behavior related to CQI; baseline empowerment was only predictor of variance in time 2 empowerment (R^2^=.62, p,.001). |
| Doran et al. ^55^ (2002)  Canada | **Effective** | Culture/ teams  To evaluate the effectiveness of an intervention designed to teach health professionals the theory and tools for the continual improvement of health care | Controlled before and after | Hospitals (4)  Multidisciplinary health care teams (10;15)  NR | **Team:** successful teams demonstrated higher baseline functional team interactions (3.4 vs. 3.15, p<.03) and problem solving effectiveness scores than unsuccessful teams (14.11 vs. 10.63, p<.05). CQI knowledge increased in both groups (time 1 to 2, p<.01; time 1 to 3, p<.02), but decreased time 3 to 4 (p<.05); intervention and controls groups improved functional group interactions (time 1 to 2, p<.001; time 1 and 3, time 1 and 3, p<.001, respectively); dysfunctional group interactions decreased in both groups (time 1 to 3, p<.001); team problem solving not related to functional team interaction or CQI knowledge. Involvement of physicians on the teams was associated with improvements in the processes or outcomes of care.  **System/clinical:** 36% of the teams were successful in implementing a change in practice that led to an improvement in the quality of care and outcomes for patients. |
| Price et al. ^49^ (2007)  Australia | **Effective** | Culture/ teams (nurse perceptions)  To identify and explore nurse managers and clinical nurses perceptions of QI as related to their practice | Qualitative, descriptive | Hospital (1)  NR  n=12 nurses | **Team:** both nurse managers and clinical nurses identified deficiencies in QI implementation that reduced its clinical impact, and neither group demonstrated responsibility for the failings nor potential improvements in the QI process. Clinical nurses: belief that QI focuses on issues related to management, and is largely irrelevant to practice. Nurse managers: reported shortsightedness, lack of knowledge, and negative attitude among clinical nurses towards QI. |
| Marsteller et al. ^54^ (2007)  USA | **Effective** | Culture/ teams (QI collaboration team interaction)    To document the interactions among teams participating in three QI collaboratives and to examine the associations between team roles in the network and performance | Descriptive | Teams from separate hospital, physician group, clinic, or health plan (94)  NR | **Team:** having large number of ties to other teams and number of times a team was mentioned as a leader were related (r=.80, p<.0008), and both were related to faculty-assessed performance (r=.31 p<.048, r=.32 p<.032, respectively). Leader nominations: trend toward relation to the number of changes the team made to improve care during the collaborative (r=.35, p=.08) and to the depth (or expected leverage) of the changes (r=.24, p=.08); number of ties (r=.49, p=.0032) and ties that featured detailed how-to advice (r=.41, p=.024) were related to depth of changes made. |
| Kollberg et al. ^59^ (2005)  Sweden | **Effective** | Culture/ teams (team outcomes on performance)  To increase the understanding of how the flow model is designed and implemented by development teams; to follow up the requirements in the National Care Guarantee through eight measures | Descriptive | Hospitals (32), county development teams (6)  NR | **System:** half of teams indicated readiness for change through the initiation of model of performance; systemic changes made: new IT ideas to support registration procedures, developing new administration systems, reviewed clinical activities, and collaboration between teams and departments changed how medical work is performed and organized. Problems: motivating employees, gathering support for the new model, implementing in clinics for internal medicine, and management taking a passive role in decision making and in supporting the flow model processes only happened in 4/6 cases. |
| Lammers et al. ^60^ (1996)  USA | **Effective** | Culture/ teams (team training)  To describe VHA sites with regard to their commitment to TQI, their activities in the areas of training, team involvement, and planning; predict how much training the site engaged in at the management and physician leadership levels, how many teams are underway, and how much total improvement occurred at the site | Descriptive | Hospitals (36)  NR  n=228 QI leaders  n=36 coordinators | **Barriers/facilitators:** commitment levels of various groups appear to be nested hierarchically: perceptions of top management commitment were independent of perceptions of lower levels of the organization. Low-commitment facilities were more than twice as likely to have reported a TQI budget (p=.013), and engage in less training of the director, associate director, chief of staff, and quality coordinator (p<.05) than moderate and high commitment sites. Age of the quality council explained 9% of the variance in number of teams; number of days of training for the leadership team explained 14% of the variance in faculty commitment; faculty, physician, and employee commitment explained between 10-15% of the variance in perceived improvement. |
| Horbar et al. ^45^ (2003)  USA | **Effective** | Culture/ teams, best practices    To develop aim for improvement in clinical, operational and organizational culture | Descriptive | Hospitals (34)  Neonatal ICU (34) | **System:** teams developed 51 potentially better practices, and each focus group developed a “resource kit” summarizing its work, many of which have been tested and implemented at the participating centers using rapid-cycle improvement. |
| Brown et al. ^57^ (2003)  USA | **Effective** | Culture/ teams, best practices  To review key elements of culture through refinement of 7 potentially better practices in one neonatal intensive care unit | Descriptive | Hospital (1)  Neonatal ICU (1)  n=215 patients | **Team:** improved ability to keep staff abreast of activities on unit, and feelings of acceptance on team; difficulty getting everyone to schedule attendance to conflict management workshop. |
| Branowicki et al. ^53^ (2001)  USA | **Effective** | Culture/ teams, clinical practice/ process  To examine and improve processes relating to the oncology patient | Descriptive | Children’s hospital (1)  NR | **Team:**  very good or excellent rated effectiveness of clinical practice committee (89%); clinical practice committee definitely improved communication between health care providers (86%); work of the clinical practice committee” definitely” resulted in system improvement and positive outcomes (81%). |
| Berenholtz et al. ^38^ (2004)  USA | **Effective** | Infectious processes (blood stream infection: catheter-related)  To eliminate catheter-related blood stream infections in the ICU, by using a QI model | Cohort | Teaching hospital (1)  ICU - surgical (1); ICU - cardiac(1)  intervention n=22,785 patient days, n=19,905 catheter days control n=21,964 patient days, n=17,383 catheter days | **System:** intervention estimated to have saved $1,945,922/year.  **Clinical:** catheter-related blood stream infection rate decreased in the intervention group (11.3/1,000 to 0/1,000 catheter days, p<.001) and not significantly in control group (5.7/1,000 to 1.6/1,000 catheter days, p=.56). |
| Berriel-Cass et al. ^19^ (2006)  USA | **Effective** | Infectious processes (catheter related blood stream infection, ventilator assisted pneumonia)  To reduce nosocomial infections using IHI model of bundles | Interrupted time series | Teaching hospitals (2 – case studies)  ICU - surgical, medical, cardiac, cardiovascular (4), and ICU -medical/ surgical (1) | **System:** average LOS decreased (8 to 4.9 days).  **Clinical:** ventilator-associated pneumonia rate decreased (8.2 to 3.3/1,000 ventilator days, p=.02); average number of days on a ventilator and total ventilator days decreased; catheter-related blood stream infection rates decreased by 55% (9.6 to 3.0/1,000 catheter days). 92% of the central lines were placed using bundles. |
| Brown et al. ^41^ (2006)  UK | **Effective** | Infectious processes (nosocomial blood stream infection)  To describe the use of a multidisciplinary approach to sepsis surveillance and evaluate impact on outcome | Case-control | Children’s hospital (1)  Pediatric ICU -cardiac /ECMO center (1)  n=215 patients | **Clinical:** sepsis decreased (24 to15 patients, p= .08); rate of sepsis decreased significantly in neonate (21% to 4%, p =.02) and pediatric respiratory patients (50% to 15%, p =.02); despite proportion of cardiac patient with open sternum decreased, the rate of sepsis (14% to 23%, p=.3) and frequency of infection increased in cardiac patients (17% to 50%, p=.02). |
| Horbar et al. ^22^ (2001)  USA | **Effective** | Infectious processes (nosocomial infection, chronic lung disease)  To make measurable improvements in the quality and cost of neonatal intensive care using a collaborative QI model | Controlled before and after | Hospitals (10;66)  Neonatal ICU (6+4;66)  intervention- infection (6 units): n=3063 patients  intervention- chronic lung disease (4 units):n=738 patients  control (66 units): n=21509 patients | **Clinical:** rate of infection with coagulase-negative staphylococcus (22.0% to 16.6%, p=.007) and nosocomial infection decreased in the infection group (26.3% to 20%, p=.007); rate of supplemental oxygen at 36 weeks’ or rate of death at 36 weeks (55.9% to 47.6%, p=.039) and adjusted gestational age decreased in the chronic lung disease group (43.5% to 31.5%, p=.03). Comparison groups coagulase negative staph decreased (15.4% to 14.5%; p=.025); nosocomial infections in intervention group larger than the comparison group (-5.6% to 1.6%; p=0.58); change in coagulase-negative staphylococcus (-5.4% to -0.8%; p=.026); death or supplemental oxygen at 36 weeks was larger in the intervention group than the comparison group (-8.3 %vs.- 1.2%, p=.14); death rate decreased (27.4% to 25.4%, p=.017). |
| Halm et al. ^39^ (2004)  USA | **Effective** | Infectious processes (pneumonia)    To evaluate an evidence-based, multifactorial intervention to improve the quality and efficiency of inpatient pneumonia care and patient understanding of their disease using the standard QI tools and resources available to the most hospitals in the United States | Cohort | Teaching hospitals (4)  NR  pre n=1013, post n=1081 patients | **System:** use of guideline-recommended antimicrobial therapy increased (78.1% to 83.4%, p=0.003); proportion of patients being discharged prior to becoming clinically stable decreased (27.0 %to 23.5%, p=.06); no improvements in time to first dose of antibiotics, proportion receiving antibiotics within 8 hours, timely switch to oral antibiotics, timely discharge, length of stay, or patient education outcomes. |
| Weiner et al. ^51^ (1997)  USA | **Effective** | Leadership  To examine if leadership for quality promote clinical involvement in hospital QI efforts, and what roles do top management, boards, and physician leaders play in promoting clinical involvement | Descriptive | University-based and non-teaching hospitals (1870)  NR | **Barriers/facilitators:** 11% of all active staff physicians had received formal QI training, and only 8% were on QI project team; senior management leadership for quality and board leadership for quality showed significant, positive relationships with measures of clinical involvement in CQI/TQM; active-staff physician involvement in governance showed positive, significant relationships with clinical involvement measures; physician-at-large involvement in governance showed significant, negative relationships. |
| Bradley et al. ^50^ (2003)  USA | **Effective** | Leadership  To define key roles/activities comprising senior management involvement in QI, develop taxonomy to classify such roles/activities, propose key elements of management involvement that might be central to success for quid; examine clinical process, use of beta blockers after AMI | Qualitative, descriptive | Hospitals (8)  NR | **Team:** personal engagement of senior leaders was paramount to success and was characterized by advocacy for QI efforts, participation in the teams, and dissemination of QI data. |
| Harris et al. ^37^ (2000)  Canada | **Effective** | Maternity (labor induction)  To improve the process of labor induction and eliminate all inappropriate inductions | Interrupted time series | Women’s hospital (1)  Delivery suite (1) | **Clinical/System:** induction rates were already declining before the project was initiated; rates continued to fall after the intervention and then leveled off; induction rates declined for all induction indicators for except fetal demise and major fetal anomaly; no change in newborn outcomes for Apgar scores less than 7 at 5 minutes, perinatal deaths, or level II/level III nursery admissions following implementation. |
| Skupski et al. ^31^ (2006)  USA | **Effective** | Maternity (major obstetric hemorrhage)  To improve outcomes of episodes of major obstetric hemorrhage. | Before and after case series | Teaching hospital (1)  NR  Total obstetric deliveries: pre n=5811, post n=12912 patients  Major obstetric hemorrhage: pre n=12, post n=49 patients | **Clinical:** increase in caesarean births (p<.001), repeat caesarean births (p=.002), and cases of major obstetric hemorrhage (p=.02); improvement in mortality due to hemorrhage (p=.036), lowest pH (p=.004), and lowest temperature (p<.001). |
| Catsambas et al. ^47^ (2002)  Zambia, Niger, Chile | **Effective** | QA projects/ evaluation  To clarify critical issues for all key aspects of QA activities; draft a guide to provide a flexible vehicle for different approaches; test and adapt the guide as it evolved in three countries; test two evaluation tools | Descriptive | National healthcare systems (3)  NR | **System:** countries and programs differ significantly in the QA activities they emphasize, how their programs evolved, and the context in which QA is implemented; management and logistics are significant factors in the success of evaluations, especially those that are coordinated overseas; the developed evaluation guide helps evaluators: agree on a proposed evaluation’s scope and design; develop an evaluation methods plan; and address QA history, advocacy, culture, and structure, as well as QA activities and accomplishments; appreciative evaluation is an approach to concurrent data collection and analysis for engaging participants more fully in the evaluation. |
| Bouchet et al. ^46^ (2002)  Zambia | **Effective** | QA projects/ evaluation  To review the performance of the Zambia Quality Assurance Program and provide recommendations to help design its next phase | Descriptive | Health facilities (24)  NR | **System:** over 5 years, senior staff built a QA structure and capacity throughout Zambia, generated enthusiasm for QA, and initiated teamwork on quality of care issues by motivated health staff. Some challenges remained: lack of integration of QA; 36% did not finish first cycle; teams did not always document data, yet 30% report significant change. |
| Pronovost et al. ^44^ (2003)  USA | **Effective** | Quality indicators    To create the idealized design of an ICU and estimate the potential impact of current performance on quality of ICU care. | Descriptive | Community teaching and non-teaching hospitals (1-pilot, 13)  ICU – surgical (2-pilot of data collection tool), ICU –urban adult med/surgical (13) | **Clinical:** respondents did not find tool burdensome; 100% of data elements were completed; K statistic=0.9 for appropriate sedation and 1.0 for each of the other measures; median percentage of days in which ventilated patients received therapies that they should: 64% for appropriate sedation, 67% for elevating head of bed, 89% for peptic ulcer disease prophylaxis, and 87% for deep venous thrombosis prophylaxis; median rate of appropriate transfusion was 33%. |
| Gandhi et al. ^48^ (2003)  USA | **Effective** | Quality indicators  To describes the OECD Health Care Quality Indicators Project, in identifying clinically meaningful and comparable measures for the quality of care in five priority areas | Descriptive | National healthcare systems (9)  NR | No results reported. |
| Thor et al. ^52^ (2004)  Sweden | **Effective** | Tool application  To understand how systematic QI efforts are established in health care organizations; understand how facilitators helped clinical teams and managers apply TQM principles and tools, and how they developed their own skills simultaneously | Descriptive | Teaching hospital (1)  NR | **Team:** clear division of labor between facilitators and other actors in the improvement efforts was an important strategy; facilitators developed specialized facilitation skill and experience in a short time, could transfer insights from improvement efforts across the organization, enable joint learning and used it to help achieve improvement; clout might help facilitators gain acceptance; facilitators developed their skills through experience, feedback, and systematic reflection. |
| Brewer ^61^ (2006)  USA | **Effective** | Errors/ adverse events (patient safety)  To test the transtheoretical integration model, which proposes relationships among team-based phenomena and patient safety and resource-use outcome variables | Descriptive | University-based and non-teaching hospitals (4)  Surgical-medical: medical, surgical, medical/surgical, telemetry, acute rehabilitation (16)  n=430 clinicians | **Team:** group-type hospital culture accounted for 30% of the explained variance of patient falls with injury; developmental-type hospital culture explained 36% of expenses per day. team concepts did not predicted falls; team design and positive/negative intrateam processes (p<.10) were predictive of longer LOS for patients on medical-surgical units |
| Brickman et al. ^26^ (1998)  USA | **Effective** | Clinical practice/ process (CAP/Stroke CHF)  To improve care and reduce costs for clinical processes related to 11 diseases with a great impact on cost/quality, high volume/cost, problem prone and/or high profile | Controlled before and after | Hospitals (4)  NR  *Case 1*: n=68;400 patients  *Case 2*: n=106;600 patients (retrospective) n=106;400 patients  *Case 3:* n=43;500 patients | **System:** decreased mortality rate (12.5 to 9.0), LOS (7.5 to 6.0 days; 6.4 days to 4.0; 9.8 day to 5.3 days), cost per case ($13,000, p<.05; $2000), and unplanned admissions.  **Clinical:** urinary tract infection decreased by 25%. |
| Houston et al. ^42^ (2003)  USA | **Effective** | Infectious processes (nosocomial pneumonia reduction)  To reduce the incidence of nosocomial pneumonia in a hospital setting | Case-control | Health care system (1)  ICU (31)  3:1 case control, 240 medical records examined | **System:** patients who developed nosocomial pneumonia demonstrated higher average LOS (31.0 vs. 12.3 days) and substantially increased total charges and mortality.  **Clinical:** 37% decrease in the pneumonia rate in the CV recovery room; able to initially capture 50% of high-risk patients after the first quarter, and increased over time to a capture rate of 80% after 1 year; 18% developed nosocomial pneumonia. |
| Mayo ^35^ (1996)  USA | **Effective** | Asthma (adult)  To assess the effectiveness of a program to improve care of adult patients hospitalized for asthma | Historically controlled | Teaching hospital (1)  NR  pre n=65, post n=61 patients | **System:** LOS for all medical service patients decreased (12.3 to 11.8 days, p<.001); LOS reduced for all patients with asthma (mean=0.8 days, 17%), and in the patients with uncomplicated asthma (mean=1.2 days, 26%).  **Clinical:** increased patient (p<.001) and house staff education (p<.001); increased use of peak flow measurements and spacers (p<.001); decreased dose and duration of IV corticosteroid administration (p<.002); patients switched to oral corticosteroids earlier, and more patients were given inhaled corticosteroids (p<.001); IV aminophylline use stopped completely; oral theophylline use declined (p<.001). |
| Fox et al. ^23^ (2006)  USA | **Effective** | Cardiac (AMI)  To improve care for patients with AMI; to improve the discharge process and thus developed discharge instruction forms, patient information booklets and resources for hospital and community | Interrupted time series | Hospitals (5)  NR | All hospitals experienced significant improvements in core measure data.  **System:** improved overall compliance score for angiotensin-converting enzyme inhibitor for left ventricular systolic dysfunction (70% to 85%); compliance for heart failure increased (68% to 77%) and was considered more successful, given the low level of initial compliance (50%). |
| Feldman et al. ^29^ (2006)  USA | **Effective** | Cardiac (heart failure)  To describe detailed information about the organizational, leadership, and cultural changes that the implementation of Project CARE; utilized national guidelines for the care of patients with acute coronary syndrome and congestive heart failure | Before and after case series | Teaching hospital (1)  NR | **System:** LOS for congestive heart failure patients (5.5 vs. 6 days in general internal medicine unit) and acute coronary syndrome was comparatively lower (4 vs. 7.5 days in internal medicine).  **Clinical:** 40% of patients had admission orders initiated from order sets post-intervention; end of year evaluations for attending contact improved (2.8 to 4.2); quality of clinical experience score increased (4 to 4.35); use of angiotensin converting enzyme inhibitors improved (87 vs. 75%). |
| Baker et al. ^43^ (2005)  USA | **Effective** | Cardiac (heart failure)  To determine whether participation in a QI collaborative for heart failure was associated with better interpersonal aspects of care and health outcomes | Descriptive | University-based and non-teaching hospitals (7;7)  NR | **System:** participants had similar quality of life but fewer emergency department visits and hospitalizations.  **Clinical:** more participants weighed themselves daily and recorded their weight (88% vs. 34%, p<.01), know how often to check their weight (p<.01), recognize symptoms of worsening heart failure (p<.01 for all), have a scale ( p<.002), monitor their weight daily (p<.001). |
| Wagner et al. ^21^ (2001)  USA | **Effective** | Clinical practice/ process (diabetes)  To improve clinical care of diabetes in 26 health care organizations | Before and after case series | Health care organizations (26)  NR | **System:** improved self-rated overall organizational function (p<.005), self-management support (p<.0004), delivery system design (p<.004), decision support (p<.007), and clinical information systems (p<.0002). All teams achieved a faculty-rated and benchmarked mean performance score of 4+.  **Clinical:** patients for whom HbA1C levels were tested twice during the past year improved (median 30 to 63%); patients with such HbA1C values improved (median 37% to 58%); patient self-management documented goals was 70% (range 37-73%), no improvement in many teams; 8/26 teams reported on the percentage of patients whose HbA1C was less than >8%; 3/8 teams decreased mean HbA1C levels for their panel of patients (median 8.5 to 7.5%). |
| Hermida & Robalino ^18^ (2002)  Ecuador | **Effective** | Maternity (maternal/child care)  To determine the effects of hospital quality assurance interventions on compliance with clinical standards, availability of essential drugs, client satisfaction, and utilization | Controlled before and after | Hospitals (4;4)  Maternity (4;4) | **System:** interventions produced rapid increases (i.e., as early as 2 months) in compliance with clinical standards, mean percent compliance higher in intervention group (97.6% vs. 74.6%, p<.0001); no differences between groups in utilization patterns or client satisfaction; intervention group demonstrated increased use of standardized list of drugs (87.1% vs. 53.6 %, p<.0001), pharmacy standardized list of drugs (94.8 %vs. 50.6 %, p<.0001), standardized clinical record for consultation intervention (62.7% vs. 22.6%, p<.0001).  **Clinical:** intervention group demonstrated higher proportion of providers who performed 12 standardized activities ( 54.6% vs.14.5%, p<.0001); regular checks (50.4% vs.17.6%, p<.0001), completed intervention (37.8 % vs. 5.8%, p<.0001), followed intervention (29.1% vs. 5.3%, p<.0001), and treated children with pneumonia in accordance with guidelines (83.7% vs. 82.2%, p=0.65); no difference in proportion of patients who can name danger signs after consultation (76.7% vs. 71.2%); no difference in increase of antenatal consultations (18.5 % vs11.2%) and outpatient consultations for sick children (21.2 % vs. 34.4%). |
| Bédard et al. ^32^ (2006)  Canada | **Effective** | Pain management  To determine if implementation of a comprehensive evidence-based program for pain management result in a significant difference in evidence-based orders, level of pain, impact of pain, satisfaction, and beliefs between patients | Historically controlled | Teaching hospital (1)  Surgery (1)  pre n=76, post n=71 patients | **Clinical:** difference in age and language between groups (p<.05); after intervention, more patients in received evidence-based orders (83% vs. 35%, p<.001), had lower pain scores, experienced fewer disturbances in sleep, walking, and general activities, were less likely to believe that good patients avoid talking about pain (F=2.92 p=.09), more inclined to believe that experience of pain as a sign that illness has become worse (F=3.11p=.08). |
| Carter & Meridy ^66^ (1996)  USA | **Patient Centered** | Patient flow/ timeliness  To the development and implementation of a performance improvement plan | Descriptive | Hospital (1)  NR | **System:** problems with the open visiting policy in ICU were remarkably infrequent; families appreciated the availability of pagers and the physical improvements. |
| Reiley et al. ^114^ (1996)  USA | **Patient Centered** | Patient flow/ timeliness (discharge)  To elicit patients and family feedback to evaluate how well patients are discharged and to help develop strategies to improve discharge planning | Before and after case series | Teaching hospital (1)  Surgery/cardiac stroke, medical, surgical/medical (4) | **Team:** continuity and transition improved (p<.05). |
| Hickey et al. ^67^ (1996)  USA | **Patient Centered** | Patient flow/ timeliness (discharge planning)    To identify discharge planning-related problems | Descriptive | Teaching hospital (1)  NR  case: post n=24, follow-up n=18 patients  control: post n=27, follow-up n=24 patients | **System:** higher proportion of patients rated discharge planning as excellent or very good after intervention (cycle 1: 86 vs. 63%; cycle 2; 83 vs. 73%). |
| Bookbinder et al. ^63^ (2005)  USA | **Patient Centered** | Clinical practice/ process (palliative care)  To improve palliative care for inpatients who are expected to die from advanced disease | Controlled before and after | Hospital (1)  Palliative care: oncology, geriatrics, inpatient palliative/ hospice (3); general medical (2)  n=156; 257 patients | **Clinical:** at baseline, patients on comparison unit more likely to have delirium and confusion (47% & 42% vs 82%p=.0001) and agitation evaluated (p<.0001). Dying patients who resided on geriatrics, oncology and palliative care/hospice units were more likely to be considered dying by the staff (95% & 66% vs 45% p<.0001) and have a comfort plan in place (90% & 46% vs 22%p<.0001), have DNR orders than the comparison units (100% & 95% vs 72% p<.0001); mean number of symptoms assessed increased significantly in all units (p<.001); number of problematic symptoms identified (p<.014) and the number of interventions consistent with PCAD increased only on the palliative care/hospice unit (p<.021); number of medical consultations declined on all units, significantly for the geriatrics and oncology units (p<.037). |
| Cleeland et al. ^64^ (2003)  USA | **Patient Centered** | Pain management  To improve delivery of pain management to VHA patients and to compare team process and patient report data on key goals from selected study units | Descriptive | Health care system (70 teams of 22 networks’ participated)  NR | **Clinical:** improved reporting of moderate or severe pain (p<.014); moderate or severe pain decreased (24% to 17%, p<.001); pain assessment increased (75% to 85%; p<.0004); pain care plans for patients with at least mild pain increased (58% to 78%; p<.0001); number of patients provided with pain educational materials increased (35% to 62%; p<.016). |
| Campese ^69^ (1996)  USA | **Patient Centered** | Pain management  To describe a pain management program implemented by perioperative nurses, using a pain level scale | Descriptive | Health care system (1)  NR | **Clinical:** pain control score was 92.1 (goal was >90). |
| Elf et al. ^68^ (2007)  Sweden | **Patient Centered** | Culture/ teams (team design of clinical space)    To explore to what extent and how the use of system dynamics contributes to the collaborative design process in the early stages of the design of a new health care environment in which various stakeholders are involved | Qualitative, descriptive, mixed methods | Hospital (1)  Stroke (1) | **System:** facilitated an explicit description of patient-centered stroke care; created dialogue and a joint understanding among disciplines. |
| Briscoe & Arthur ^65^ (1998)  USA | **Patient Centered** | Culture/ teams (satisfaction: customer, physician, employee), clinical practice/ processes  To improve customer, physician and employee satisfaction | Descriptive | General hospital (1)  Surgical, medical (2) | **System:** some actions showed immediate results; spin-off projects. |
| Frush et al. ^115^ (2006)  USA | **Safe** | Errors/ adverse events (patient safety)  To review the design and implementation of a comprehensive patient safety program; describe some of the barriers that impedes the development of a culture of safety. describe safety program development and implementation related to the practice of radiology | Descriptive | Health care system, university-based teaching hospital (1)  NR | **System:** voluntary reports of medical errors increased (600 to 1100). |
| Weeks et al. ^71^ (2001)  USA | **Safe** | Errors/ adverse events (medication)    To reduce medication errors within the VA system | Before and after case series | Collaborative – teams (27)  NR  n= 54,000 patients over the project  pre n=1833, post n=1866 errors averted | **System:** teams reported improvements on at least one marker of ADE (78%); reported sustained improvement (52%). Faculty-rated high performance was correlated with team in involvement in pre-collaborative conference calls (r=.398, p<.04), team completion of early requests for documented changes (r=.518, p<.006), team reporting of learning new ideas in the first learning session (r=.488, p<.014). Teams collectively averted 1833 medication errors, and an additional 1866 at follow-up. Teams which reported that they learning new ideas during the learning sessions were 12.8 times more likely to maintain gains (p=.018); teams that learned new methods of QI were 24.8 times more likely to apply those methods in different physical locations (p=.002), and 24 times more likely to apply those methods to different topics (p=.002); teams that continued to collect data were 30 times more likely to maintain gains (p=.001). Potential direct-care cost saving (range $3.47-12.13 million). |
| Sim & Joyner ^76^ (2002)  USA | **Safe** | Errors/ adverse events (medication)    To improve medication safety, with a focus on work undertaken to reduce medication variance | Descriptive | Hospital (1)  NR | **System:** twofold increase in reporting in first seven months, which then leveled off. |
| Silver & Antonow ^12^ (2000)  USA | **Safe** | Errors/ adverse events (medication)    To modify hospital medication systems to reduce the incidence of medication errors | Before and after case series | University-based and non-teaching hospitals (13/39)  NR | **System:** professional self-report: reduction in error frequency (26.9%), administration errors (19.4%), transcription/ verification errors (35.7%); increases in error discovery/ prevention increased (12.5%, p=.032), overall reporting (11.4%, p=NS), reporting of errors that reached the patients (24.1%, p=.013). |
| Costello et al. ^70^ (2007)  USA | **Safe** | Errors/ adverse events (medication)  To increase medication-error reporting and reduce the severity of medication errors reported in the pediatric critical care center by implementing a pediatrics medication safety team | Before and after case series | Children’s hospital (1)  Pediatric ICU (1)  n=109 medication errors | **System:** medication-error reporting increased; error severity decreased; reporting of near-miss errors increased (9% to 38% to 51%). |
| Cimino et al. ^72^ (2004)  USA | **Safe** | Errors/ adverse events (medication)  To establish a methodology, generalisable across a broad range of settings, for identifying, documenting, analyzing, and reporting prescribing errors; determine the overall rate of prescribing errors (i.e., benchmark) among participating pediatric hospital PICUs; report the relative effectiveness of a variety of hospital-specific, self-selected interventions in reducing medication errors and ADEs in PICUs | Before and after case series | Children’s hospital, hospital (9)  Pediatric ICU (9)  n=12,026 medication records | **System:** at least one prescribing error in baseline orders decreased (11.1% to 7.6%, p<.001); rate of incomplete orders decreased (18.7% to 13.8%, p<.001); interception of prescribing errors improved (1.6% to 2.0%, p<.01); non-intercepted orders decreased (2.0% to 0.8%, p<.01); preventable ADEs were uncommon (0.13% to 0.03%, p<.05). Sites that reported a patient physician ratio of <=4 had a mean medication error rate of 12.3% compared with a mean rate of 37.5% for sites having a ratio >4 (p<.01). |
| Carey & Teeters ^74^ (1995)  USA | **Safe** | Errors/ adverse events (medication)  To assess efforts to reduce medication errors | Interrupted time series | General hospital (1)  NR  n=420,000 errors | **System:** IV doses omitted decreased (LCL 10.4 np 25.5 UCL 40.6); training module was not effective in the reducing the number of wrong IV doses (LCL =0, np 7.3, UCL 15.5). |
| Adachi & Lodolce ^73^ (2005)  USA | **Safe** | Errors/ adverse events (medication)  To describe FMEA and its application to prevent dosing and administration errors with IV medications | Before and after case series | Hospital (1)  NR | **System:** wrong-dose decreased (59 to 46); 3/7 error types decreased: iv-pump (24 to 10), epidural-pump (3 to 1), concentration (7 to 0). |
| Frankel et al. ^80^ (2003)  USA | **Safe** | Errors/ adverse events (medical error)  To create a common patient safety strategy a large, integrated, non-profit health care delivery system | Descriptive | Health care system (1)  NR | Leadership felt that the rounds had great value. |
| Hasler et al. ^77^ (2001)  USA | **Safe** | Errors/ adverse events    To develop a model for determining error | Descriptive : case study | Teaching hospital (1)  ICU – medical, emergency, (2) | **System:** all reviewers agreed that the probability of secondary adrenal insufficiency was high enough to warrant steroids; proper treatment with stress-dose steroids may have increased the chance that she would have lived to obtain surgery; thus, this failed treatment was an error |
| Rask et al. ^79^ (2006)  USA | **Safe** | Errors/ adverse events (patient safety)    To describe the design elements that were particularly important for sustaining the Partnership for Health and Accountability program, along with the partnerships experience with member participation in reporting activity and patients’ safety improvement processes | Before and after case series | Hospitals (148), hospitals (2 - case studies 315 beds; 53 beds)  NR | **System:** 88% of hospitals provided data for joint commission core measures, even when their scores did not fall in the top 50%; 97% of hospitals publicly reported their data (97%); 69.1% of hospitals that begun projects in 2002-03 were successful in reducing the incidence of targeted errors; the mean reduction in 2004 was 34%. |
| Allison & Toy ^81^ (1996)  USA | **Safe** | Errors/ adverse events (blood donation)  To improve blood availability, in response to a rapid rise in incident reports and complaints for physicians, nurses, patients, and families | Interrupted time series | Teaching hospital (1)  Blood donation unit (1) | **System:** number of incident reports reduced (19 to 2); donations without prior physician orders decreased (50% to 9%); patients without medical record numbers decreased (153 to 18/quarter). |
| Apkon et al. ^75^ (2004)  USA | **Safe** | Errors/ adverse events (medication)  To examine the impact of process changes on the reliability of delivering drug infusions using FMEA; improve patient safety, efficiency in staff workflow, hemodynamic stability during infusion changes, efficient use of resources | Descriptive | Teaching hospital (1)  Pediatric ICU (1)  n=10 nurses | **System:** 1500 fewer infusions prepared by nurses per year; all nurses perceived that system changes were safer. |
| Korytkowski et al. ^82^ (2006)  USA | **Safe** | Clinical practice/ process (glycemic management)  To reduce the frequency and adverse outcomes caused by severe hypoglycemia in the inpatient population | Descriptive | Teaching hospital (1)  NR | **System:** form was used for 63% of orders after 2 months, and 91% after 1 year; 10-fold reduction in prescribing errors; incidence of hypoglycemia did not change, but hyperglycemia declined over time. |
| Farbstein & Clough ^78^ (2001)  USA | **Safe** | Errors/ adverse events (medication)  To describe a medication reliability project, based on 16 best practices, of a six hospital collaborative. | Before and after case series | University-based and non-teaching hospitals (6)  NR | **System:** sound-alike/ lookalike errors decreased; PCA incidents occurred less frequently (once every 13.8 to 24.4 days); one error occurred in Coumadin administration after intervention; education of patients increased (79% to 100%); frequency of nurse verification with patients about medication increased (79% to nearly 100% to 90%); rationale-stating improved (63% to 75% to 90%); process backlog reduced (by 45 minutes).  **Clinical:** fraction of patients who faster receipt of therapeutic anticoagulation increased (44% to 93%, p<.001); reached therapeutic level from 16-31% and out of range levels decreased (69% to 47%). |
| Bluth et al. ^87^ (1993)  USA | **Timely** | Patient flow/ timeliness (waits)    To solve a common radiology problem: delays in the timely interpretation of preoperative chest radiographs. | Before and after case series | Teaching hospital (1)  Radiology (1) | **System:** chest radiographs interpreted before surgery improved (83% to 98%, p<.001), waiting period between patient check-in at the outpatient reception area and interpretation of the preoperative chest radiograph reduced (275 to 77 minutes, 72% reduction, p<.030). |
| Alberts et al. ^86^ (1999)  Sweden | **Timely** | Patient flow/ timeliness (treatment delay)  To shorten the time to make a diagnosis and to begin definitive treatment of severely injured patients | Before and after case series | Teaching hospital (1)  Trauma (1)  pre n=23, post n=38 patients | **Clinical:** immediate medical care delayed/inappropriate decreased (9/23 vs. 2/38 patients, p=.001); time needed to make a diagnosis was <4 hours for all patients; time needed to start definitive treatment of head injuries <4 hours increased (9/12 vs. 18/21 patients); internal fixation of femoral fractures started within 4 hours increased (2/11 vs. 12/17 patients). |
| Berry et al. ^85^ (1998)  USA | **Timely** | Patient flow/ timeliness (heparin administration)  To design and implement an anticoagulation treatment guideline across the entire integrated delivery system for deep venous thrombosis or pulmonary embolus | Historically controlled | Hospitals (4)  NR  pre n= 192, post n=222 patients | **System:** time to first APTT of >50 seconds improved (73% to 95%, p<.001). |
| Gilutz et al. ^91^ (1998)  Israel | **Timely** | Cardiac (AMI thrombolytic therapy delay)  To identify and address causes of delays in thrombolytic therapy in patients arriving at a high volume emergency department with AMI and thereby reduce the “door-to-needle time” | Historically controlled | Hospital (1)  ICU – cardiac, emergency (2)  pre n=40, post n=33, peri n=27, follow up n=32 | **Clinical:** mean door-to-needle-time decreased (61.8 to 47.6 minutes, p<.029); prolonged door-to-needle-time time intervals of the late versus the early groups was primarily due to extended decision-making time (36.0 vs. 13.6 minutes, p< 0.003), followed by time to therapy initiation (26.2 vs. 11.1 minutes, p<.002); door to needle time for early versus late pre, peri, and post times all significant (p<.001). |
| Horbar et al. ^83^ (2004)  USA | **Timely** | Patient flow/ timeliness (time to surfactant administration)    To test whether teams in neonatal intensive care units exposed to a multifaceted collaborative QI intervention based on four key habits would administer the first dose of surfactant sooner after birth, and achieve improved patient outcomes for preterm infants of 23-29 weeks’ gestation | Randomized controlled (cluster) | University-based and non-teaching hospitals (57;57)  Neonatal ICU (57); neonatal ICU (57) | **Clinical:** intervention hospitals more likely than control hospitals to receive surfactant in the delivery room (adjusted odds ratio 5.38 [95% CI 2.84 to 10.20]), less likely to receive the first dose more than two hours after birth (adjusted odds ratio 0.35 [95% CI 0.24 to 0.53]), received the first dose of surfactant sooner after birth (median of 21 vs. 78 minutes, p< .001); mortality and pneumothorax (NS).  Chronic lung: rate of supplemental oxygen declined: 55.9% to 47.6% (p=.039), comparison 1994-1996 and comparison NICU: infection rate at comparison decreased 22.6 % to 21,1 %p=.002, staph infection from 15.4% to 14.5% (p=.025) other infections 10.3 % to 8.95% (p<.002) staph infections at 6 infection subgroup NICU (-5.4% vs. -8% p=.026) ; death or supplemental oxygen rate of oxygen post conception 54.5% to 53.3% (p=.09) oxygen alive 36 weeks (36.4 vs. 36.3 p=.57) death rate 27.4% to 25.4% (p=0.17) additional analysis from 1994 to 1997 the majority of which were significant. |
| Gering et al. ^89^ (2005)  USA | **Timely** | Coordination (inpatient consolidation)  To integrate its two acute medical surgical hospitals | Before and after case series | Hospitals (2)  NR | **System:** cancellations in OR decreased (16.4% to 5.5%, p<.001); average number of monthly scheduled appointments and clinical wait times (NS). |
| Kallenbach & Rosenblum ^97^ (2000)  USA | **Timely** | Cardiac (procedures: carotid endarterectomy)  To achieve a standardized, coordinated 24-hr stay in an intermediate step-down unit | Before and after case series | General hospital (1)  ICU/ surgery, medicine, intermediate care (3) | **System:** 1 readmission occurred on postoperative day 21 for new-onset pneumonia; mean LOS decreased (3.24 to 2.13 days), charges decreased by 20.6%**.**  **Clinical:** no inpatient workups were delayed (first 2 quarters post-implementation). 6/40 patients were not discharged on postoperative day 1: 2/6 returned to surgery, 4/6 patients each experienced new-onset atrial fibrillation, fever, nausea, and need for transesophageal echocardiography. |
| Tunick et al. ^90^ (1997)  USA | **Timely** | Cardiac (procedures: CV surgery)  To redesign the entire process of caring for patients – from referral to discharge – on the CV surgery service | Before and after case series | Teaching hospital (1)  Surgery - CV(1) | **System:** average waiting time for transfer decreased (9 to 0 hours), preoperative LOS decreased to 0 for many patients; same-day admissions increased (> 300%); number of patients discharged <7 days increased (5% to 17%); number of outliers (LOS > 21 days) decreased (25 %to 19%). |
| Gall ^88^ (1996)  USA | **Timely** | Patient flow/ timeliness (admission, discharge)    To improve the admission/discharge process | Before and after case series | Teaching hospital (1)  General medical floors (2) | **System:** decreased bed turnaround by 50%, waiting time, complaints, staff frustration; reduced number of calls between units by 60%, number of beds left over from first shift by 90%, number calls from nursing for stat beds. |
| Bartlett et al. ^84^ (2002)  Australia | **Timely** | Patient flow/ timeliness (waits), patient satisfaction    To bring together 17 major emergency departments to reduce both clinical and operational waits and delays and to improve patient satisfaction | Interrupted time series | Hospitals (18=17 emergencies + 1 hospital)  Emergency (17) | **General:** all teams achieved a score of >3.5, one-third scored >4.5; 32/47 clinical projects were completed, with 31 resulting in significant improvement or achieving target; 30/39 operational projects were completed, with 24 of these achieving improvement or target; all hospitals recorded significant improvements in analgesia time. |
| Benson & Harp ^92^ (1994)  USA | **Timely** | Patient flow/ timeliness (waits)    To reduce emergency department waiting times, using systems thinking tools | Descriptive | Hospital (1)  Emergency (1) | **System:** reduced waiting times (19%), reduced variation in waiting time by 67 minutes, reduced patient complaints (28%), increased physician satisfaction. |
| Cooperative Cardiovascular Project ^94^ (1998)  USA | **Timely** | Cardiac (AMI care)  To describe exemplary QI plans, relating to effective intervention strategies for AMI care. | Descriptive | Hospitals (36 interviewed, 191 data collection)  NR | **System:** LOS decreased (6%), time to patient treatment in ER improved (3%), expansion of cardiac program (3%)  **Clinical:** increased usage of beta-blockers (3%), increased QI knowledge/capacity (physician and multidisciplinary interaction (22%), stimulated pathway creation (6%). |
| Yancer et al. ^98^ (2006)  USA | **Timely** | Patient flow/ timeliness (overcrowding, capacity)    To alleviate overcrowding and increase hospital capacity | Before and after case series | Hospital (1)  Emergency (1) | **System:** Diversion hours reduced (2365 to 655 hours, 72% reduction); ALOS reduced (3.87 to 3.69 to 3.61), ALOS for admitted patients decreased by 25 minutes; improved patients satisfaction (3.96 to 4.11); monthly average of number of patients boarding in the ED decreased (190 to 120). |
| Isouard ^96^ (1999)  Australia | **Timely** | Patient flow/ timeliness (resource utilization)    To examine the key elements that were used to develop a TQM environment for improving pathology services | Descriptive | Teaching hospital (1)  Pathology services (1) | **System:** improvements for all five pathology service improvement programs. |
| Heilig ^95^ (1990)  USA | **Timely** | Patient flow/ timeliness (resource utilization)    To describe an organizational-wide quality enhancement process | Descriptive | Teaching hospital (1)  NR | **System:** average waiting time decreased; complaints about waits have declined (50 to fewer than 10 per month); savings of more than $200,000 in admitting costs; increased the number of patients by 33%, decreased cancellations by 25%; waiting time for patients for surgery decreased by 50%; saved $500,000 a year in unnecessary stays; reduction in patient length of stay after being judged ready to leave; reduction in inquiries to patient accounts. |
| Carboneau ^93^ (1999)  USA | **Timely** | Culture/ teams (team time management)  To accelerate improvement efforts through team time management | Descriptive | Hospital (1)  Rehabilitation services (1) | **System:** rehabilitation services scheduling team, patients staffing teams, and other hospital teams have been successful in accomplishing their goals within 6 months; reduced the cycle time for QI efforts by 75%. |
| Hobde et al. ^99^ (1997)  USA | **Efficient** | Coordination (hospital, clinical, operational)    To reduce overall operational and clinical process costs | Descriptive | Teaching hospital (1)  NR | **System/clinical outcomes:** operational efficiency team: $190000 in cost reductions; interventional cardiology team: LOS decreased in 4 units, complication occurrence decreased (5.5 to 3%). |
| Isouard ^111^ (1999)  Australia | **Efficient** | Cardiac (AMI, clinically indicated tests)  To assess the effect of a TQM multidisciplinary approach on the appropriateness of clinical laboratory testing of patients with AMI | Controlled before and after | Teaching hospital (1;1)  Clinical pathology services (1)  intervention: pre n=253, post n=252 patients  control: pre n=211, post n=203 patients | **System:** proportion of clinically indicated tests requested increased (77.5 %to 88.2%, p<.01), number of non-clinically indicated tests reduced (81.7%, p< 0.01). |
| Ziegenfuss et al. ^109^ (1998)  USA | **Efficient** | Patient flow/ timeliness (resource utilization)  To maintain/enhance patients care, improve the quality of working life, and to do so in a budget-neutral manner | Descriptive | Teaching hospital (1)  Pediatrics, medicine, surgery (3) | **General Outcomes:** 60% of initiatives had positive outcomes, 18% had mixed outcomes, 16% had not identified outcomes, and 6% had negative outcomes. |
| Cholewka ^110^ (1999)  Lithuania | **Efficient** | Patient flow/ timeliness (resource utilization)  To presents an overview of a hospital's strategy, initial steps, and main accomplishments, as well as mitigating factors that arose in its quest to manage its own resources in response to new political, economic, and social environments | Descriptive | Teaching hospital (1)  NR | No outcomes reported. |
| Walley & Gowland ^107^ (2004)  UK | **Efficient** | Patient flow/ timeliness (emergency care improvement)  To evaluates the use of PDSA improvement cycles; to report on improvement roles played by senior management and clinicians, response of senior managers and clinicians to incremental improvement activities, and relationships between roles and improvement outcomes | Descriptive | Healthcare regions (10)  Emergency (12) | **General**: many senior professionals still misinterpret how PDSA cycles should work; teams that only implemented PD in PDSA cycle did not measured rigorously and front line staffs were not aware of reason for change; in teams that implemented the full PDSA cycle, managers were used as a resource, and front line staff conducted the majority of work. |
| Dugar ^106^ (1995)  USA | **Efficient** | Cardiac (AMI)  To describe how a small hospital implemented CQI without the use of a consultant | Descriptive | Hospital (1)  NR | **System:** desired results were not met after 1 year; with an estimated overall cost to implement the 5-year plan ($10,000); communication and working relationships amongst departments improved; compliance rate decreased in all areas being measured at 6-month follow-up. |
| Mazur et al. ^105^ (1996)  USA | **Efficient** | Asthma (pediatric)  To identify variation in treatment practices to streamline patients care and determine the most cost-effective interventions; to reduce variation in patients care and costs by developing a pathway for management of asthma in children | Before and after case series | Teaching hospital (1)  NR  n=70 charts reviewed | **System:** average LOS decreased (2.6 to 2.2 days), average total cost for those patients decreased ($3761 to $2440). |
| Blackburn & Neaton ^103^ (1997)  USA | **Efficient** | Cardiac (procedures: carotid endarterectomy)  To redesign the care for patients having CEA surgery | Before and after case series | Hospital (1)  Surgery - vascular (1)  n=185 patients | **System:** difference in major complications; LOS decreased by 1.15 days; costs reduced by $1900 per case, with no change in postoperative morbidity or mortality rates. |
| Eavy & Conlon ^100^ (1993)    USA | **Efficient** | Coordination (IV supply - HC/supplier team, product implementation)  To improve product quality | Descriptive | Health care system (1)  NR | **System:** 91% of nurses thought new product better than previous; annual cost savings of $172,000. |
| Wang et al. ^112^ (2003)  Taiwan | **Efficient** | Culture/ teams  To explore members' job satisfaction, morale, organizational commitment, and inventory management through QI team intervention in a department of anesthesiology | Controlled before and after | General hospital (1)  Anesthesiology (1); operating room (1)  intervention: n=45 nurses  control: n= 50 nurses | **System:** job satisfaction improved month (month 4 p=.019, month 8 p=.003); organizational commitment improved at month 8 (p=.034); monthly consumable material stock decreased in intervention group and increased in control group. |
| Clemmer et al. ^102^ (1999)  USA | **Efficient** | Patient flow/ timeliness (resource utilization), patient care  To change both the thinking and practice patterns in the shock/ trauma/ respiratory intensive care unit; to reduce costs | Before and after case series | Teaching hospital (1)  ICU - shock/ trauma/ respiratory (1) | **System:** severity of illness increased (p<.0001); mortality increased (p<.0001), but NS after controlling for admission severity, mortality rates NS when analyzed mortality by severity; unadjusted hospital and shock/ trauma/ respiratory ICU LOS increased (p<.03), but NS after controlling for severity. After controlled for severity, cost centers directly affected by care delivered ICU personnel and in total costs decreased, compared with baseline (p<.05). Cost reductions for medical surgical ICU (p<.045) and in the emergency department (p<.0001). |
| Sanborn et al. ^108^ (1996)  USA | **Efficient** | Patient flow/ timeliness (resource utilization)    To improve use and reduce costs of 5-HT3 antiemetic agents | Before and after case series | Teaching hospital (1)  Oncology, hematology, pediatric oncology/hematology (3) | **System:** average drug cost per patient decreased 35%; monthly expenditures on 5-HT3 agents decreased; 5-HT3 expenditures as a function of costs for chemotherapy patient days decreased; treatment failures with these agents were reduced (NS). |
| Curley et al. ^101^ (1998)  USA | **Efficient** | Patient flow/ timeliness (resource utilization)  staff satisfaction  To improve the care of patients on inpatient wards | Randomized controlled | Teaching hospital (1)  Inpatient wards (3;3)  intervention: n= 567 admissions  control: n=535 admissions | **System:** mean LOS lower (5.46 vs. 6.06 days, p=.006), mean total charges lower ($6,681 vs. $8,090, p=.002); provider satisfaction: greater understanding of patient care, more effective communication, and more teamwork in intervention group (each p<.006); compliance: intervention group higher than control group in respiratory therapy (91.7% vs. 73.6%, p=.075) and nutrition services (100% vs. 80%, p=.018). |
| Alemi et al. ^113^ (2001)  USA | **Efficient** | QA projects/ evaluation  To describe a variety of improvement efforts and their impact on the organizations that sponsored them | Descriptive | Health care organizations (32) and teams (92)  NR | **System:** 41/92 projects with start-end dates; 79% of 88 projects collected data to examine improvements (mean= 62 days); sampling and planning for data collection occurred with 17% of 66 projects; 76% of 70 targeted client satisfaction with services (11 successful, 32% improvement); 21% focused on improving sales/market share (7 projects reported average of 28% improvement); 56 of projects reported improved patient outcomes; 46 projects improved employees work life. Most projects did not intend to save costs, and of those that did were unsuccessful. |
| Beesley et al. ^104^ (1993)  USA | **Efficient** | Patient flow/ timeliness (resource utilization)    To implement QI techniques to improve laboratory services in a hospital | Before and after case series | Teaching hospital (1)  Laboratory, blood services (2) | **System:** Increased employee accountability; process improvements (laboratory medicine); increased customer and employee satisfaction; cost savings (departmental - $75,000). |
